# What’s New in Traumatic Brain Injury: Update on Tracking, Monitoring and Treatment

**DOI:** 10.3390/ijms160611903

**Published:** 2015-05-26

**Authors:** Cesar Reis, Yuechun Wang, Onat Akyol, Wing Mann Ho, Richard Applegate II, Gary Stier, Robert Martin, John H. Zhang

**Affiliations:** 1 Department of Anesthesiology, Loma Linda University Medical Center, Loma Linda, CA 92354, USA; E-Mails: cesarreis@hotmail.com (C.R.); rapplegate@llu.edu (R.A.); gstier@llu.edu (G.S.); rdmartin@llu.edu (R.M.); 2 Department of Physiology and Pharmacology, Loma Linda University School of Medicine, 11041 Campus Street, Risley Hall, Room 219, Loma Linda, CA 92354, USA; E-Mails: monkeymei2@aliyun.com (Y.W.); onatakyol@hotmail.com (O.A.); wingiho@googlemail.com (W.M.H.); 3 Department of Physiology, School of Medicine, University of Jinan, Guangzhou 250012, China; 4 Department of Neurosurgery, University Hospital Innsbruck, Tyrol 6020, Austria; 5 Department of Neurosurgery, Loma Linda University School of Medicine, Loma Linda, CA 92354, USA

**Keywords:** traumatic brain injury, neuroimaging, electrophysiology, stem cell, hypothermia, nano particle, biomarker, microdialysis, proteomic, genomic

## Abstract

Traumatic brain injury (TBI), defined as an alteration in brain functions caused by an external force, is responsible for high morbidity and mortality around the world. It is important to identify and treat TBI victims as early as possible. Tracking and monitoring TBI with neuroimaging technologies, including functional magnetic resonance imaging (fMRI), diffusion tensor imaging (DTI), positron emission tomography (PET), and high definition fiber tracking (HDFT) show increasing sensitivity and specificity. Classical electrophysiological monitoring, together with newly established brain-on-chip, cerebral microdialysis techniques, both benefit TBI. First generation molecular biomarkers, based on genomic and proteomic changes following TBI, have proven effective and economical. It is conceivable that TBI-specific biomarkers will be developed with the combination of systems biology and bioinformation strategies. Advances in treatment of TBI include stem cell-based and nanotechnology-based therapy, physical and pharmaceutical interventions and also new use in TBI for approved drugs which all present favorable promise in preventing and reversing TBI.

## 1. Introduction

Traumatic brain injury (TBI) is characterized as “an alteration in brain function, or other evidence of brain pathology, caused by an external force” [1] and is present in all societies as the most severe, disabling neurological disorder for more than 57 million patients worldwide [2]. TBI occurrence peaks during both youth and later in life, and is one of the most common causes of morbidity and mortality of young adults less than 45 years of age [3]. The World Health Organization anticipates that it will be ranked as a dominant cause of death and disability by the year 2020 [4]. Furthermore, it is estimated that approximately 1.7 million people experience TBI each year in the United States [5]. In Europe, estimated annual health care cost exceeds 33 billion euros for this type of injury [6]. Among those injured, many individuals will have physical disability leading not only to a burden on the health care system but more importantly, functional impairments impeding quality of life [7,8,9]. TBI can also lead to outcomes of physical inactivity such as obesity, diabetes, and heart disease [10,11,12,13]. It is quite imperative to trace, monitor and treat TBI more efficiently with the aid of advanced technologies and multiple therapeutic strategies. In this review, we shall systematically summarize the related progress in these aspects with emphasis on updated information.

It wasn’t until 1976 that Graham Teasdale and Bryan J. Jennett came up with a method to objectively diagnose unconsciousness, as TBI may be associated with loss of consciousness. They established the Glasgow Coma Scale (GCS) [14]. With the GCS, rapid assessment of possible brain pathologies can be done by measuring spontaneous and stimulated verbal, motor or eye opening responses, thus allowing evaluation of best response. The length of unconsciousness dictates the severity of brain injury. 

Mild, moderate and severe TBIs are classified according to GCS scores of 13–15, 9–12, and 3–8, respectively. Severe injury causes not only permanent neurological deficits (20% of adults) but it has been shown that up to 14% remain in a vegetative state and 20%–40% of patients end up dying as a result of brain injury or secondary complications [2]. Over the past two decades, several studies demonstrated that a significant amount of central nervous system damage occurring after TBI resulted from secondary brain injuries [1,2,3,4], which involves a complex cascade of biochemical events eventually leading to delayed tissue damage and cell death [5,6].

Conventional neuroimaging methods, like CT and MRI, show advantages in detecting secondary injuries. However, newly developed structural and functional neuroimaging techniques such as diffusion-tensor-imaging, magnetoencephalography and single-photon-emission-computed-tomography improve accuracy and timeliness of diagnosis and prognosis in TBI. We will introduce and compare the most current methods used in clinical settings. Compared to advanced neuroimaging technologies, detecting molecular biomarkers in biofluid like blood, cerebrospinal fluid, urine, saliva, *etc.* is more feasible and effective in diagnosis, prognosis and treatment guidance of TBI. At the genomic and proteomic levels, modern bioinformation helps to explore more specific and sensitive TBI-restricted biomarkers for early diagnosis and long-term prognostication. Multimodal neuromonitoring technology has been shown to play an important role in treatment of TBI, preventing secondary brain injury and promoting better patient outcome. Here we shall introduce the traditional monitoring methods including electrophysiologic monitoring, brain oxygen monitoring, intracranial pressure (ICP) monitoring, cerebral microdialysis and experimental brain-on-chip technology.

For the treatment of TBI, we shall focus on updated information about stem-cell based and nanotechnology-based therapies like perfluoro-carbons and polyethylene-glycol-functionalized hydrophilic-carbon-clusters and also cover the physical and pharmaceutical interventions like hypothermia and hyperbaric oxygen preconditioning. New uses in TBI treatment for approved drugs like progesterone and lithium are also discussed.

## 2. Gene Expression Profiles after TBI

Advances in technology include but are not limited to: single-nucleotide polymorphisms, global gene expression approach, microarray techniques, mass spectrometry, wide genomic, transcriptomic, proteomic and epigenomic profiling approaches, gene interaction hierarchy, and Ingenuity Pathway Analysis program. Through these technologies, a substantial amount of genetic factors have proven to be implicated in the pathogenesis of brain trauma. Evaluating the influence of polymorphisms on TBI helps to better understand the individual variations in outcomes, aids in the triaging and management of TBI patients, and ultimately contributes to genetic profile-based personalized interventions [7].

### 2.1. Genetic Polymorphisms Influence Recovery

Clinical outcomes following TBI are determined by both multiple genetic elements and acquired environmental risk factors. TBI triggers a series of pathophysiological processes including neuroinflammation, oxidative stress, excitotoxicity, apoptotic cell death, neurodegeneration, reparative processes, synaptic plasticity, and neurotransmitter alterations [7,8]. Additionally, genetic factors have been implicated in almost all these processes to some extent and are therefore responsible for the variable individual responses to TBI [7,8,9]. An individual’s genetic predisposition to the injury may influence the variability of the initial response, the recovery process, susceptibility to secondary injury, and response to rehabilitation.

Genetic association studies are useful tools in investigating possible relationships between gene polymorphisms and disease outcome. With the microarray techniques, Michael and colleagues [10] detected 5000 gene expressions in brain tissue from four TBI subjects, one subject with vacuities, and one subject with normal brain tissue. They found that 1200 gene segments increased their expressions and 104 transcripts showed differential expressions. The candidate genes affecting TBI outcomes include apolipoprotein E (APOE), p53, angiotensin I-converting enzyme (ACE), D2 subtype of the dopamine receptor (DRD2), atechol-*O*-methyltransferase (COMT), BCL-2, neuroglobin (NGB) and IL-1β [11]. Genetic susceptibility to clinical outcomes after TBI was reported in recent clinical studies, as the genetic loci showed close link with the clinical phenotype including polymorphisms in APOE, brain-derived neurotrophic factor (BDNF), endothelialnitric oxide (NOS3), monoamine oxidase A (MAO-A), interleukin 6 (IL-6), neurofilament heavy (NEFH), serotonin transporter protein (SLC6A4), COMT, calcineurincatalytic isoform A-gamma subunit (PPP3CC), and kidney and brain expressed protein (KIBRA) genes [8].

Previous studies investigated genetic variants involved in various molecular pathways initiated after TBI, including oxidative stress, neuroinflammation, excitotoxicity, apoptotic cell death, neurodegeneration, reparative processes, synaptic plasticity, and neurotransmitter alterations [7,8,9]. Given the multiple biological processes involved in the pathogenesis of neurotrauma, the majority of genetic factors that influence TBI outcomes remain unknown. There may be several other relevant genes awaiting identification, and detection of these genes requires a combination of wide genomic, transcriptomic, proteomic and epigenomic-profiling approaches [8,12].

Gene expression and epigenetic-epigenomic approaches studies have been conducted on nonhuman TBI models to understand the pathophysiology and recovery process after TBI. With a global gene expression approach, Di Pietro and colleagues found that gene expression regulation in rat hippocampus was determined by the severity of injury, and the associated pathways that were altered the most were those involved in inflammation, immunity, and neurodegeneration [13]. A candidate gene study in mouse hippocampal tissue also showed activation of inflammation pathways after TBI, with up-regulation of inflammatory genes starting at the site of injury, spreading throughout the hippocampus, and thus preceding the onset of secondary injury [12,14].

### 2.2. Local and Remote Gene Expression Profiles

Bioinformatic analysis tools allow for both identification of key molecules and elucidation of their interactions. It made the utilization of microarray data more practical and easily replicable. Based on a database built from published scientific literature, Ingenuity Pathway Analysis (IPA) software is designed to depict direct and indirect gene interactions and to assign genes to specific biological functions and canonical pathways [8]. When compared to the non-injured control brain, the information of local and remote gene expression profiles will provide us with valuable insight into delayed neuronal injury as well as endogenous neuroprotective mechanisms. White and colleagues utilized the IPA program to analyze the pathways and networks related to TBI-induced genetic alterations. They first examined gene expression on both sides of the brain; data showed that a robust proinflammatory response was associated with TBI in ipsilateral brain tissues. Interestingly, the contralateral side of the brain also exhibited significant gene expression changes after TBI, which suggested a remote anti-inflammatory response. Furthermore, they used multiple network and pathway approaches to identify genes of interest (GOI) and revealed two distinct pathways associated with post-TBI secondary neural injury. They include toll-like receptor/NF-κB signaling pathway and JAK/STAT signaling pathway. These genes of interest identified molecules and molecular pathways in response to TBI and could provide novel targets for therapeutic strategies [15].

### 2.3. miRNAs Expression Profiles

miRNAs are small RNA (~22 nts) molecules that are expressed intrinsically and play an key role in regulating gene expression. miRNAs have proven to be important mediators of the massive molecular and cellular changes that occur in both short and long-term periods post TBI. In a study of rodent TBI models, deep sequencing identified eight upregulated and thirteen downregulated miRNAs at 24 h post-injury, whereas three were upregulated and thirteen downregulated at day 7 post-injury [16]. Microarray analyses in rat models of TBI revealed dynamic temporal regulation of miRNA expression within the cortex, with the numbers of downregulated and upregulated miRNAs peaking at 24 h and 72 h post-injury respectively [17,18]. In the mouse hippocampus, 50 miRNAs were decreased, whereas thirty-five miRNAs were increased after TBI [19]. Bioinformatic analysis of the predicted targets for validated miRNAs regulated by TBI (miR-107, -130a, -223, -292-5p, -433-3p, -451, -541, and -711) revealed an overrepresentation of proteins involved in several biological processes and functions initiated after injury, including signal transduction, transcriptional regulation, proliferation, and differentiation. Redell and colleagues further observed how miR-21 was upregulated in hippocampus, particularly in the dentate gyrus and CA3 region in a TBI model [20]. Expression of cell death protein 4 as a target of miR-21 decreased in these regions, denoting a potential survival mechanism mediated by miR-21in TBI-affected cells [21].

Mechanical force is one of important determinants in TBI, and Weber and colleagues proposed how mechanical shear stresses could modulate vascular homeostasis through microRNA regulation of target molecules, like phosphatase and tensin homologprotein (PTEN) [22]. They conducted a study on human endothelial cells subjected to unidirectional shear stress, and data showed 13 microRNAs with increased expressions; among them miR-21 was expressed the most. miR-21 targets phosphatase and PTEN. Further studies in human vascular endothelial cells exposed to shear stress or transfected with miR-21 showed downregulation of PTEN, whereas cells overexpressing miR-21 had decreased apoptosis, increased phosphorylation of endothelial nitric oxide synthase, and nitric oxide production [22]. Together, they concluded shear stress could regulate microRNA expression in endothelial cells, thereby suggesting potential roles of microRNAs on mechanotransduction and injury response [23]. In addition Redell and colleagues [24], firstly reported plasma miRNA levels altered in TBI patients compared to age-, gender-, and race-matched healthy volunteers. Based on quantitative analysis of RT-PCR data, they suggested that plasma miR-16, miR-92a, and miR-765 be used as early circulating markers which will be discussed more detail in the subsequent section.

## 3. Biochemical Markers for TBI Diagnosis, Prognosis and Management

TBI accounts for one third of all injury-related deaths [25]. Seventy-five percent of TBIs are concussions or other forms of mild TBI. For severe and moderate TBI, neuroimaging and electrophysiological modalities as biomarkers are being routinely used in the clinical setting to help TBI diagnosis and prognosis, but for mild and chronic TBI, the molecular biomarkers detected in biofluids like blood serum, urine, saliva, cerebrospinal fluid (CSF), and extracellular fluid (ECF) are more practical and economical. Sensitive and specific biomarkers reflecting brain injuries, repair, and regeneration can provide important information regarding TBI pathophysiology and can serve as candidate markers for predicting abnormal computed tomography findings in mild TBI [26].

### 3.1. miRNAs as Diagnostic and Prognostic Biomarkers

As discussed above, miRNAs expression pattern altered significantly in response to TBI, and miRNAs play key roles in signal transduction, transcriptional regulation, mechanotransduction, proliferation, and differentiation initiated by TBI. These altered miRNAs thus seem to be good candidate biomarkers for TBI. Redell and colleagues compared the plasma miRNA profiles between severe TBI patients (Glasgow Coma Scale ≤ 8) and healthy volunteers. They concluded that decreases in the levels of miR-16 and miR-92a, and increased levels of miR-765, were good markers of severe TBI at 25–48 h after injury [27]. A simplified technique for miRNA measurement in biofluids could provide rapid diagnosis and allow for efficient treatment decisions [28].

Studies of CSF from TBI rats found a significant increase in levels of miR-let-7i as early as 3 h post-injury [29]. Prediction analysis revealed that miR-let-7i targets S100B and UCH-L1 which are TBI-related proteins, suggesting a possible role for miR-let-7i in regulating TBI pathology [30]. Three other miRNAs—miR-16, miR-92a, and miR-765 have been identified to serve as diagnostic biomarkers for severe brain injury in TBI patients. Plasma levels of miR-16 and miR-92a are also increased notably in patients with mild TBI [24]. These studies highlighted how the miRNA-based biomarkers in the serum and CSF could serve as promising diagnostic tools in TBI [18].

In addition, miRNA levels also change following TBI treatment, suggesting the use of miRNA as a prognostic biomarker. As will be discussed in the treatment section, hypothermia is one of the most promising treatments for TBI due to its ability to attenuate prolonged neurological deficits and improve functional outcomes [31,32,33]. Interestingly, some miRNAs that show altered expression after TBI are also sensitive to temperature change. In a rat fluid-percussion model of TBI, the levels of many upregulated miRNAs after TBI decreased under hypothermic conditions [34]. Among the miRNAs, miR-9 is predicted to reduce expression of proteins known to interact with actin-binding proteins and basal plasma membranes, indicating that an increase in miR-9 levels after TBI would disrupt cytoskeleton and cell-adhesion properties, thereby increasing cell death. Therefore, decreased expression of miR-9 may enhanced cell survival and help in explaining the treatment efficacy of hypothermia therapy [18].

### 3.2. Serum Autoantibodies as Long-Term Biomarkers

The permeability of the blood-brain-barrier (BBB) is increased following TBI and results in the exposure of brain-specific proteins (potential antigens) and their break down products (BDPs) to peripheral circulation immune system components, allowing the self antigen/immune cell interaction to activate the immune response post TBI. In the case of TBI, neuronal and glial cells at and around damage sites release brain-derived cell-type-specific proteins into the peripheral blood stream, leading to a subsequent increase in the formation of autoantibodies against these cells [35]. Several antigens and their corresponding autoantibodies have been identified and characterized. Here we highlight the potential use of autoantibodies as candidate blood biomarkers due to their long-term presence in serum compared to their counterpart antigens.

The presence of these autoantibodies may represent putative long-term biomarkers possibly correlated to injury severity as well disease prognostic indicators that may be the center for potential neurotherapeutic targets (see Table 1). It is conceivable that assessing the levels of these autoantibodies in combination with other markers may yield valuable clinical information; however, further studies are necessary to correlate the pattern of expression against clinical outcomes at different time points post-TBI.

**Table 1:** Auto-antibodies as diagnostic marker and/or prognostic factor in traumatic brain injuries (TBI).

Serum Auto-Antibodies	Significance	Reference
Anti-GFAP and GFAP break down products (BDPs)	Strong diagnostic marker and prognostic factor	Zhang *et al.* 2014 [36]
Anti-BMP (basic myelinprotein)	Weak diagnostic marker and prognostic factor	Ngankam *et al.* 2011 [37]
Anti-PL (phospholipid)	Weak diagnostic marker and prognostic factor	Ngankam *et al.* 2011 [37]
Anti-NMDA and Anti-AMPA	Moderate prognostic factor	Goryunova *et al.* 2007 [38]

### 3.3. Early Generation Biomarkers

Clinical proteomics aim to identify suitable biomarkers for diagnosis or prognosis of a disease. The current available protein and gene biomarkers are regarded as early generation biomarkers and in some regards show low specificities and sensitivities. These early generation biomarkers, however, are well studied and some of them have already been applied in clinical practice. We will give a brief overview on them. Representative biomarkers are derived from acute neuronal, axonal, astroglial and endothelial injuries or secondary inflammatory and reparative processes such as inflammation, oxidative stress, excitotoxicity, and other host-derived pathophysiological mechanisms [39]. In particular, early biomarkers of structural damage, such as S-100B, GFAP, and UCH-L1 may be used to assist physicians in assessing a brain injury and determining whether to order a head CT scan for patient with a mild TBI. Markers become abnormal days or weeks after injury and could be used to predict prolonged complications or to monitor recovery. Accumulating amounts of molecular biomarkers have been found to be associated with TBI outcome and recovery. In Table 2, we list the biomarkers derived from damaged structure of neurons, glial cells and endothelial cells. For the numerous other biomarkers involving inflammatory reactions, excitotoxicity, oxidative reactions *etc.* related reviews are recommended [40].

**Table 2:** Potential molecular biomarkers in TBI.

Candidate Marker	Marker Origins	Attributes	References
pNF-H (Phosphorylated Neurofilament H)	Neuron	Neuronal injury Blood levels of pNF-H levels showed significant correlations with the level of consciousness and CT findings	Ghonemi *et al.*, 2013 [41]
NSE (neuron-specific enolase)	Neuron	Neuronal injury Neuron-specific Elevated blood NSE levels have been linked to poor outcome in severe and mild TBI	Topolovec-Vranic *et al.*, 2011 [42]
SBDP150/SBDP145 (spectrin breakdown products) (Calpain-generated) αII-spectrin proteolysis	Axons and presynaptic terminals	Acute necrosis High level is associated with worse Glasgow Coma Scale (GCS), longer ICP elevation, and poor outcome following TBI	Mondello *et al.*, 2010 [43]
SBDP120 (caspase-3-generated)	Axons and presynaptic terminals	Delayed apoptosis Underlying cell death mechanisms	Brophy *et al.*, 2009 [27]
UCH-L1 (ubiquitin carboxyl-terminalhydrolase-L1)	Neuronal cell body	Neuronal cell body injury sensitive and specific biomarker elevated UCH-L1 levels are associated with lower Glasgow Coma Scale (GCS) and poor outcome after TBI	Papa *et al.*, 2012 [44]
MAP2 (microtubule-associated protein 2)	Dendrites	Dendritic injury	Kobeissy *et al.*, 2006 [45]
MBP (myelin basic protein)	Oligoden-drocytes/Schwann cells	Demyelination Excellent specificity, limited sensitivity	Berger *et al.*, 2005 [46]
S100B (Calcium-Binding Protein B) and its isoforms s100A1B and s100BB	Glia cells	Glial injury Elevated blood and urine levels of s100b, 100A1B and s100BB are associated with poor outcome in TBI	Rodriguez *et al.*, 2012 [47]
GFAP (glialfibrillary acidic protein)	Glia cells	Glial injury CNS-specificity Elevated blood GFAP levels to predict TBI outcome	Vos *et al.*, 2010 [48]
GFAP breakdown products (GFAP-BDP)	Glia cells	Specific marker of brain damage GFAPBDP >0.68 lg/L within 24 h of injury was associated with acute traumatic lesions on the CT and with unfavorable 6-month outcome	Okonkwo *et al.*, 2013 [49]
Angiopoietins-1/2(Ang-1/2)	Endothelia cells	Vascular injury and regeneration evaluation in plasma, serum and cerebrospinal fluid	Chittiboina *et al.*, 2013 [50]

### 3.4. Clinical Limitations and Outlook

Though numerous candidate biomarkers have proven a positive association with TBI outcome, they showed low specificity or sensitivity when used individually. Combining biomarkers into a panel may provide more information than individual biomarkers [51]. In a study of 206 subjects, GFAP and UCH-L1 together had better sensitivity and specificity to discriminate between TBI patients and healthy controls than either biomarker alone. The combination also had better sensitivity and specificity for predicting 3-month outcomes [52]. Other combinations of biomarkers found to have prognostic value for TBI outcomes include GFAP, UCH-L1, SBDP145 [53], NSE, and s100b [51,54].

The progressive increase in TBI brings with it more variable and complex long-term neurological disorders. The heterogenetic and multifactorial nature of secondary responses in TBI make it difficult to find cellular biomarkers for diagnosis, prognosis and management of TBI. It is imperative to find more accurate TBI-specific markers to take the place of CNS-restricted markers. Systems biology strategies can be applied to probe and analyze the crosstalk between different molecular signaling pathways that regulate the secondary cellular response through computational models, which integrate the diverse data sets. Feala and colleagues described opportunities for applying this methodology to existing TBI data sets to identify new biomarker candidates and gain insights about the underlying molecular mechanisms of TBI response. For instance, they manually compiled a list of 32 protein biomarker candidates from the literature, and then applied network and pathway analysis to recover known TBI-associated mechanisms and produce new hypothetical biomarker candidates. The combination of neuroproteomics with systems biology and other molecular biology techniques will be ideal tools for discovering well-validated TBI-specific biomarkers [55].

Although great progress has been made with the aid of advanced technologies, the need for transitioning from research-based experimental screening with mass spectrometry to fast and reliable diagnostic instrumentation is imperative for the use of biomarkers in the clinical setting.

## 4. Neuroimaging Advances for TBI Diagnosis and Prognosis

The essential aims of neuroimaging after TBI are to assist in prevention of secondary damage, to emphasize favorable biomarkers with potential for neurodegenerative disease detection, and to obtain prognostic information about long-term outcome. Neuroimaging methods such as computerized tomography (CT) and magnetic resonance imaging (MRI) which are identified as conventional neuroimaging techniques and are generally used for the initial clinical evaluation and acute vital management of intracranial problems following TBI [56]. CT is generally the imaging modality of choice for initial screening in order to exclude serious intracranial injury and provide an early assessment of the extent of brain injury [57]. MRI is indicated in acute TBI when neurologic examination is not consistent with CT findings and provides more sensitivity with detection of white matter abnormalities [58].

Diffuse axonal injury (DAI) determines long-term cognitive and neuropsychiatric outcomes following mild TBI, both hemorrhagic and non-hemorrhagic. Shearing axonal lesions are located in the major white matter tracts, spreading from the surface to deeper structures [59]; however, conventional neuroimaging methods are insensitive to DAI.

Advanced structural neuroimaging techniques are susceptibility-weighted-imaging (SWI), diffusion-weighted-imaging (DWI), diffusion-tensor-imaging (DTI) and high-definition-fiber-tractography (HDFT). Advanced functional neuroimaging methods include MR spectroscopy, functional MR imaging, SPECT and magnetoencephalography [56].

These techniques are beneficial for finding abnormalities in the brain related to long-term traumatic sequelae and the focus of ongoing research is to identify structural and functional correlations while analyzing fiber tracts [60]. The *American Journal of Radiology* in 2015 concurred that advanced neuroimaging techniques show promising results in group comparison analyses; however, there is still lack of evidence supporting the routine clinical use of advanced neuroimaging for diagnosis and/or prognostication at the individual patient level [61].

Listed below (Table 3) are the advanced structural and functional neuroimaging techniques and their association with main clinical applications and limitations.

**Table 3:** Neuroimaging.

Neuroimaging Techniques	Attributes	Limitation
Susceptibility-weighted imaging (SWI)	Microbleeding in diffuse axonal injury	Long acquisition time and sensitivity to motion artifacts
Diffusion-weighted imaging (DWI)	Non hemorrhagic diffuse axonal injury	Heterogeneity with large standard deviation of the ADC changes
Diffusion tensor imaging (DTI)	White matter integrity	FA measurement is compromised by interstitial fluid content
High definition fiber tractography (HDFT)	Structural brain connectivity	Restricted ability to determine crossing of fibers within a voxel
Functional MRI (fMRI)	Neuronal activity with cerebral oxygen consumption	Physics based factors of signal and the field inhomogeneity generated by deoxyhemoglobin
Magnetoencephalography (MEG)	Magnetic fields of postsynaptic ionic currents	Variety of incomparable approaches and absence of standard analyzing protocols
Magnetic resonance spectroscopy (MRS)	Intracellular neuronal metabolic status	Limited spatiotemporal resolution and small brain fields are challenging to analyze
Single-photon emission computed tomography (SPECT)	Regional cerebral blood flow	Regional cerebral blood flow changes after TBI do not always correspond to metabolism

### 4.1. Susceptibility-Weighted Imaging (SWI)

Susceptibility-Weighted Imaging (SWI) combines both magnitude and phase data to increase the contrast between hemorrhage-related blood products and the surrounding brain parenchyma, thereby allowing usefulness in detection of small amounts of altered blood and blood products on neuroimaging [62]. Glushakova and colleagues conducted an experimental TBI study about pathophysiological cascades, including microvascular damage and inflammation which indicated that the development of focal microbleeds was associated with delayed and localized brain blood barrier (BBB) breakdown, as well as progressive degeneration of white matter up to 3 months following injury. Delayed white matter damage and development of focal microbleeds were co-localized with cellular markers of regional inflammation [63,64]. An important advantage of SWI is the detection of microbleeding, as it suggests the presence of diffuse axonal injury and is not seen on conventional MRI [65]. Hemorrhagic lesions illustrate shear injury to small vessels and are thought to be a marker of axonal damage, but MRI may more directly reflect axonal damage of non-hemorrhagic white matter foci after TBI [66]. SWI is capable of visualizing venous blood vessel continuity, and is six times more sensitive than conventional MRI for detecting diffuse axonal injuries [67], and microbleeds. Furthermore, the total amount, volume, and extent of SWI-identified microbleeds correlate with initial Glasgow Coma Scale scores, duration of coma, and long-term outcomes measured at 6–12 months after injury [68].

A study by Ryan and colleagues aimed to investigate the utility of SWI as a biomarker for prediction of outcome and recovery of social cognition after pediatric TBI, and concluded that SWI in the acute phase of injury may be a useful indicator of not just the severity of brain injury, but may also assist clinicians to identify children at elevated risk for poor social cognitive function in the post-acute recovery period [69].

Shaken baby syndrome, now called abusive head trauma [70] have great benefits from a variety of imaging techniques. Even though studies have proposed that clinical and histological proof of this condition are related to hypoxic–ischemic injury, diffuse axonal damage seems to be an important factor [71]. Gleckman and colleagues described DAI to be the reason for mortality in almost all of their studied abusive head trauma victims [72]. Additionally, Niwa T. and collegues reported hemorrhage detection in cortical laminar necrosis with SWI due to the same syndrome [73]. This condition can have cerebral ischemia manifestation acutely as one of the essential parameters to its diagnosis, making DWI an extremely useful technique, as confirmed by Biousse V. and collegues in a study that showed DWI abnormalities in patients with the same diagnosis. These abnormalities were either diffuse or predominantly in the distribution of the posterior watershed territories [74].

Earlier studies that used MRI imaging to detect traumatic microbleeding failed to demonstrate correlation with functional outcomes [75]. SWI enabled improvements in outcome prediction in lesions that have a particularly high prognostic significance. Magnetic field strength, in-plane spatial resolution, and the distance between adjacent slices [76] may significantly affect the number of observable microbleeds, as higher spatial resolution increases microbleeding detection [77].

Wang Xuan and colleagues studied SWI combined with conventional MRI and CT images to prospectively analyze mild TBI patients 1 year post TBI. Their results showed SWI detected microbleeds that went undetected on conventional MRI and CT images. Microbleed lesions detected by SWI on the frontal, parietal, and temporal lobes may correlate with higher incidence of depression after TBI [66].

### 4.2. Diffusion-Weighted Imaging (DWI)

Diffusion-Weighted Imaging (DWI) with its known functions such as measuring patterns of water diffusion based on surrounding temperature, structures, and tissue damage [78]. Areas with a high degree of diffusion such as the cerebrospinal fluid will be hypointense on DWI and display a high ADC value [79]. Areas where diffusion is restricted due to the presence of swollen ischemic cells in acute diffuse axonal injury lesions appear hyperintense on DWI and display low ADC value [80]. Water diffusion develops over an uncertain timeline in traumatic axonal injury, likely due to the variable pathophysiological changes that occur in the intracellular compartment and may persist up to 18 days after TBI [77]. Cytotoxic edema is associated with cellular swelling by decreasing extracellular fluid and leads to intramyelinic accumulation of water, producing myelin edema [81]. In contrast, vasogenic edema is caused by increased permeability of the blood–brain barrier as well as water diffusion [82]. Unlike SWI, DWI has the ability to explain the pathophysiology of non-hemorrhagic diffuse axonal injury. White matter apparent diffusion coefficient is particularly useful for predicting outcomes [83], as it was demonstrated that values of diffusion in the white matter and in the corpus callosum correlate with functional outcomes in severe TBI patients at the time of hospital discharge [84].

Reduced ADC values in the corticospinal tracts and corpus callosum have also been observed in concussed athletes compared to controls. Galloway and colleagues attempted to predict TBI outcomes using DWI and reported that apparent diffusion coefficient values of both specific regions and the entire brain could be used as an outcome predictor in pediatric cases of severe TBI [85]. Hudak and colleagues conducted a clinical trial regarding connections between types of edema with DWI and prognosis, and reported that the cytotoxic edema group had more severe injuries and acute signs of clinical impairment when compared to participants with predominantly vasogenic edema, despite the fact that both cytotoxic and vasogenic edema groups reached a similar level of functional recovery by 6 months post-injury [86]. In an experimental TBI model in rabbits, decreased ADC values during the acute phase in the focal lesion area correlated with poor functional outcomes at 30 days. This suggests these values are associated with the extent of brain damage and have predictive value [81,82]. Non-hemorrhagic lesion load detected in the acute setting by DWI is a significant parameter, and Moen and colleagues demonstrated that the number of DWI lesions in the corpus callosum and brain stem significantly predicted outcomes for patients with severe TBI in multi-variable analyses. In addition, they investigated possible traumatic axonal injury in normal-appearing corpus callosum and its effect on outcomes. No differences between TBI patients and healthy control subjects were found. A special finding by Moen and colleagues was that the mean ADC values of the normal-appearing corpus callosum had a wide variation in the early stages, as the patient group had both very low and high ADC values, explained by the reflecting differences in pathology of injury [87].

In the early stages of TBI, diffusion of water molecules can be affected by axotomy and myelin degradation associated not only with increased ADC values but also post-traumatic cytotoxic edema with reduced ADC values [88,89]. The balance between cytotoxic and vasogenic edema generates a wide range of ADC values [90].

The fact that ADC values are high in the subacute phase indicates a massive increase in the extracellular water content of the tissue, reflecting the progressive loss of proteins in the extracellular matrix combined with cellular degeneration and increased diffusion of water molecules [91,92]. Håberg and colleagues correlated the extent of vasogenic brain edema with high ADC values in the perifocal lesion area caused by BBB breakdown [93]. Continuously higher ADC values following TBI is associated with persistent injury in the perifocal lesion area. However, partial recovery of ADC values in the perifocal lesion area during the subacute phase might be detected and explains how the complexity of physiological and molecular interactions affect their values [94]. Although DWI has important benefits in identifying axonal injury induced by trauma, the disadvantages of spatial resolution of current diffusion imaging methods and their sensitivity to signal loss from edema and hemorrhage make it difficult to visualize the specific locations of axonal injury and isolate the affected pathways. Maegele and colleagues conducted a study with experimental TBI in the acute (6 h), subacute (48 h), and chronic phase (7 days) in rats by DWI and ADC mapping and correlated these results with ATP-specific bioluminescence imaging and histology. They reported no homogeneous area of ADC alteration between both hemispheres in any of the selected regions and also extreme ADC heterogeneity with large standard deviation of the ADC changes in all regions studied. In some cases, variable ADC values in neighboring slices of the same animal were detected. This leads us to conclude that the heterogeneous ADC patterns provides a mixed expression of vasogenic and cytotoxic edema following TBI [95].

### 4.3. Diffusion Tensor Imaging (DTI)

Diffusion imaging methods have clinically influenced the improvement of TBI as a result of their ability to detect the effects of injury upon white matter structural integrity [96]. Diffusion tensor imaging (DTI), an extension of DWI, measures diffusion of water molecules within the white matter of the brain [97]. Apparent diffusion coefficient (ADC) or mean diffusivity (MD) describes a measure of the rate of diffusion of water molecules, which is usually averaged over all directions. Their values are similar when obtained from the same scanner. High fractional anisotropy (FA) and low ADC/MD values indicate that the cortical white matter tracts are intact; low FA and high ADC/MD values indicate that a nerve fiber is injured [98]. In pathological states such as edema, axonal degeneration, demyelination or fiber disruption, the diffusion of water along the direction of the nerve microstructure is affected and the amount of water diffusion increases as there are fewer intact structures binding water molecules, leading to a decrease in fractional anisotropy because of restricted axoplasmic flow and increased flow across the axonal membrane [99].

Hberg and colleagues in 2014 conducted a detailed clinical research study with 78 healthy and 73 chronic TBI patients. He outlined not only injury mechanisms, but also neuroimaging and clinical measures from the acute and early phases after injury and its impact on white matter FA, MD, and tract volumes in the chronic phase of TBI, and how white matter integrity in the chronic phase is associated with different outcome measures. They concluded that FA was the primary determinant of injury severity and mechanism, and diffuse axonal injury of any grade led to lower FA in the thalamus and brainstem. Minor effects on MD were observed in severe TBI associated with low cerebral perfusion pressure (below 70 mmHg).

Studies have shown FA several standard deviations below a control group average reflects the number of regions with “abnormally” low white matter integrity, and correlates with measures of trauma severity and cognitive dysfunction [100]. A number of areas with affected white matter, rather than the magnitude of damage within any single region, appears as the most important aspect of the brain damage associated with TBI. This is due to the specific regions of exposed white matter integrity diversified across individuals [101].

FA has been proposed as the most feasible biomarker of traumatic axonal injury and one of the best indicators of TBI severity. The most powerful applications of DTI techniques involves the specific analysis of particular regions of interest which are selected based on known or suspected tendency to demonstrate a particular pathological state [102]. In addition, sports-related mild TBI studies in older adolescents and young adults in the post-acute period [103,104] and adult mild TBI outcome studies [105] show consistent findings. Roberts and colleagues reported a meta-analysis on 20 studies in non-penetrating pediatric TBI and concluded a decrease in the integrity of white matter microstructure occurs three or more months after mild to severe pediatric TBI, and supported DTI as providing a useful biomarker of injury [106]. Most DTI analyses show a loss of structural integrity in acute symptomatic TBI patients who presented decreased FA values in widespread regions, especially located in frontal lobe, limbic system, and sublobar areas compared with healthy controls [107]. In a cohort of 30 adults admitted for neurorehabilitation, Sidaros and colleagues found that FA was decreased globally in the white matter of patients compared to controls, and the cerebral peduncle FA correlated with the Glasgow outcome score at 1 year following injury [108]. Messe and colleagues using DTI, showed a statistically significant difference in mean diffusivity within the long association white matter tracts between patients with good and poor outcomes based on their symptoms [109]. Dodd and colleagues reported a meta-analysis on 31 studies published from 2002 to 2013 assessing white matter injury during the acute/semi-acute phase of mTBI, and DTI results show decreased FA in chronic TBI and in more severely injured TBI cohorts. Mixed findings were found in the semi-acute (less than 3 months post-injury) phase of mTBI. Multiple inflammation mechanisms might potentially explain these diffusion variations in DTI [110]. Cubon and colleagues suggested that mean diffusivity (MD) may be more sensitive than FA to effects of mTBI [111]. However, Davenport and colleagues did not detect any result indicating greater sensitivity for FA than MD [112].

### 4.4. High Definition Fiber Tractography (HDFT)

High Definition Fiber Tractography (HDFT), tracks fibers from the cortex to subcortical targets with high resolution [113]. It also reveals more than 250,000 fibers with 256 possible directions, enabling it to project complex 3D anatomies and superior resolution of the white matter compared with standard DTI [97,114].

HDFT applies diffusion spectrum imaging, which has a high angular resolution than release tractography to navigate complex fiber crossings. Thus, HDFT provides high-resolution details of axonal pathways and projection fields that allow exposure of specific location and degree of damage [85]. Shin and colleagues reported a use of HDFT to identify white matter damage following TBI in a case where a 32-year-old man sustained a severe TBI with Glasgow Coma Scale score of 6 and left/right asymmetries. They identified a specific lesion of the corticospinal pathway in the corona radiata that was precisely correlated with the functional deficits. The volumetric losses as well as the number of fibers projecting from the motor cortices were diminished in the injured side, corresponding to the left upper-extremity weakness. In addition, despite basal ganglia hemorrhage present on imaging methods such as CT, structural MRI, and DTI, the patient’s hemiplegia could not be entirely explained by basal ganglia damage. With HDFT, they specified lesions in the corticospinal pathway adjacent to the basal ganglia and quantified the extent beyond the capabilities of other neuroimaging modalities, which suggested damage in the posterior limb of the internal capsule. Due to analysis of HDFT outcomes, high-resolution white matter imaging and comparisons of left/right fiber tractography supports significant diagnostic information in characterizing the nature of motor pathologies [115].

### 4.5. Functional MRI (fMRI)

Functional MRI (fMRI) assesses brain function based on relations between neuronal activity and the resultant hemodynamic responses described as neurovascular coupling [116,117]. fMRI studies generally focus on not only cognitive and behavioral sequelae of TBI but also episodic memory circuitry activation after trauma, post-traumatic stress disorder, and major depressive disorder following blast injury [118]. Functional deficits in speeded information processing and attention can be detected after frontal lobe damage following TBI [119].

fMRI is usually performed while the patient completes a neurocognitive task, placing an increased demand on the brain, thus showing differences in local perfusion in real time. Abnormal brain activation patterns detected by fMRI correlate with a broad range of neurocognitive and functional deficits in patients with prior traumatic coma, including memory impairment and motor dysfunction [120]. In a cohort of adolescents with chronic moderate-to-severe TBI, patients performing a perspective-taking task exhibited BOLD signal changes in regions such as the cuneus and parahippocampal gyrus that were not observed in controls. Differential fMRI brain activation patterns were present despite the fact that patients exhibited normal performance scores on neurocognitive tests [121]. These results suggest that recovery of neurocognitive function might concern the enrollment of additional neuroanatomic connections [122]. Positive correlations between self-reported measures of symptom severity and increased activation of both dorsolateral and ventrolateral prefrontal cortex suggests potential compensatory activation [123]. Talavage at al., conducted an evoked fMRI study that also utilized a pre- *vs.* post-injury design in athletes. Their results indicated that prolonged exposure to sub-concussive hits resulted in hypoactivation within the left middle and superior temporal gyri, left middle occipital gyrus, and bilateral cerebellum during a working memory task, which correlated with decreased working memory performance in the non-concussed high school football players [124].

Resting-state fMRI (RS-fMRI), a recent form of fMRI, is based on the analysis of spontaneous low-frequency fluctuations in the BOLD signal in the absence of any external task. Thereby, it focuses on functionally related brain regions at rest [125]. One of the most studied resting state brain networks is the default mode network (DMN) [126]. DMN describes a state of consciousness in which an individual is awake and alert, but not actively participating in any cognitive or functional tasks [127].

Nathan and colleagues designed a descriptive study that aimed to understand how functional connectivity of the resting state DMN varies between healthy controls and mTBI subjects differs. They reported that mTBI patients had a decrease in DMN connectivity within regions of the left inferior temporal lobe, right precentral gyrus, and left caudate nucleus. They also demonstrated significant correlations between a variety of neuropsychological test measures and data from DMN connectivity maps of TBI subjects in cerebellar and cortical regions. Their results show the ability to correlate markers of emotional response within the cerebellum. fMRI is sensitive to changes in neural function following TBI and is an effective technique for understanding the neural basis of cognitive-behavioral dysfunction and investigating recovery of function [128].

### 4.6. Magnetoencephalography (MEG)

Magnetoencephalography (MEG) detects magnetic fields generated by currents flowing in neurons through a group of sensors surrounding the head [129,130]. Abnormal delta-waves emerge from deafferentation of neurons following traumatic axonal injury in brain tissues after TBI to the white matter fiber tracts, as demonstrated on diffusion tensor imaging with reduced fractional anisotropy [131]. The patient repeatedly performs tasks such as auditory, visual, tactile or electrical stimuli while MEG detector documents the brain activity [132]. MEG can examine neuronal change and reorganization during normal interactions between different brain regions as exemplified in language processing [133] or abnormal connections between multiple suspected epileptogenic regions following TBI [134].

Huang and colleagues detected sensitivity of MEG for analyzing resting-state MEG data and found abnormal delta-waves in 84.5% for all mild and symptomatic TBI patients [135]. Multiple regions in the frontal lobe, posterior parietal lobe, inferior temporal lobes, hippocampus, and cerebellum have a relatively higher likelihood for generating abnormal MEG slow-waves than other brain areas, indicating that these regions are also particularly vulnerable to head trauma [131]. A recent MEG study in a group mixing mild, moderate, and severe TBI patients showed reduced functional connectivity primarily in bilateral frontal regions with left parieto-temporo-occipital regions greater than right [136].

The role of MEG in diagnosis and prognosis is still limited because of the variety of incomparable approaches and absence of standard protocols for MEG analysis. MEG applications require improvements before becoming clinically relevant. More studies are needed to examine the utility of MEG as a prognostic and predictive neuroimaging method for TBI.

### 4.7. Magnetic Resonance Spectroscopy (MRS)

Magnetic Resonance Spectroscopy (MRS), a molecular-based neuroimaging technique that reflects intracellular metabolic status as evidence of microscopic injury [137]. Common neuronal markers utilized during MRS include: N-acetylaspartate (NAA), a neuronal mitochondrial marker that decreases with neuronal loss or dysfunction; creatine (Cr), a marker for intact brain energy metabolism; and choline (Cho), a marker for membrane disruption, synthesis or repair. Increasing amounts of Cho detected in white matter is a breakdown product after myelin damage [138].

Metabolites such as lactate (Lac) (generated as a result of anaerobic glycolysis) and glutamate (excitatory amino transmitter) might suggest evidence of energy dysfunction, membrane turnover, and inflammation as a result of injury [139]. The relative concentrations of metabolites are described as an array of peaks along a spectrum; abnormal ratios between the peaks prove metabolic dysfunction following TBI. Neuronal metabolites measured with MRS allows direct detection of the brain region of interest [61]. Harris and colleagues conducted an experimental rat study to describe neurometabolic effects of TBI using high field MRS. They reported alterations of 20 metabolites in the rat brain following TBI. MRS scans were performed 2 weeks before TBI and up to 14 days after TBI. Functional deficits, lesion characteristics, and neurochemical profiles of TBI in contused and normal-appearing brain regions were evaluated. Neurometabolic changes at the cortical contusion site included fourteen neurochemicals significantly decreased and five (Gln, PCho, Lac, Ala, and Ser) increased after TBI [140].

Various studies indicate that NAA levels and NAA/creatinine ratios are reduced in TBI as a result of neuronal loss and/or dysfunction. Reduced NAA levels are predictive of long-term functional outcomes in TBI. Twelve athletes who had sustained sports-related concussions were compared with healthy controls within 6 days of injury. The NAA/Cr levels were significantly reduced in the primary motor cortex and, to a lesser extent, in the dorsolateral prefrontal cortex when compared to healthy controls [141]. Sports-related concussions and NAA/Cr relationships were studied by Vagnozzi and colleagues in 14 sports related concussed athletes. MRS was performed at 3 to 4 days, 15 days, and 30 days after injury. Decreased NAA/Cr ratio was detected at 3 to 4 days following concussion and limited recovery was seen at 15 days post-injury. NAA/Cr ratio was within a normal range at 30 days postinjury. Despite abnormal MRS findings, postconcussive symptoms resolved at 3 days following TBI [142]. Maudsley and colleagues studied 40 closed-head TBI patients who underwent DTI and MRS. Changes of both DTI and MRS following TBI are widely distributed and predominate across white-matter regions, with the metabolic changes seen on MRS covering a wider spatial extent than those seen in the DTI. These changes also reflect different aspects of the injury, considering extensive metabolic changes can be seen despite absence of measured DTI changes [143]. Tollard and colleagues performed MRS on 43 patients 24 days post TBI. The study aimed to predict functional outcomes at one year. They found that NAA/Cr ratios-specifically in the pons, thalamus, and insula-predicted an unfavorable 1-year outcome with 75% sensitivity and 75% specificity. Furthermore, when MRS was combined with DTI measurements, the combined predictive value of MRS and DTI for unfavorable outcome increased to 86% sensitivity and 97% specificity [144].

Govind and colleagues conducted a study during the subacute period following TBI. They studied not only the associations of metabolite indices with neuropsychological test results, but also differences in metabolite ratio distributions. Hemispheric lobes and tissue types were evaluated relative to those of age-matched controls. Significant metabolite alterations were present in locations remote from, or in the absence of any MRI-observed traumatic lesions, and significant correlations were found between metabolite measures [143].

### 4.8. Single-Photon Emission Computed Tomography (SPECT)

Brain perfusion single-photon emission computed tomography (SPECT) imaging is a functional nuclear imaging technique performed to assess regional cerebral perfusion by using 99 mTc ethyl cysteinate diethylester (ECD) as tracer [145].

SPECT scans can display areas of impaired brain function following TBI including not only focal cerebral blood flow (CBF) reduction near the focal site of injury, but also asymmetrical hypoperfusion in the prefrontal, temporal, and parietal or occipital lobes. In addition, a decreased CBF in the anterior temporal poles was observed [146,147]. SPECT discloses regions of hypoperfusion correlated with severity of acute injury, loss of consciousness, cognitive deficit, and brain atrophy [148].

Some early studies compared the value of CBF SPECT imaging to anatomical findings on MRI or CT in TBI patients. Ichise and colleagues [146] conducted a correlational study and reported that SPECT imaging was able to detect abnormalities in 66% of patients who had chronic symptoms following TBI, whereas MRI and CT did so in 45% and 34% of 29 patients, respectively [146]. Similarly, Kant and colleagues reported abnormal SPECT scans in 53% of patients, while MRI and CT detected 9% and 5% of 43 patients with persistent post-concussive symptoms, respectively [149]. Newberg and colleagues have demonstrated significant cerebral blood flow differences in right frontal lobe, and left parietal lobe of patients who reported headaches post-TBI [150]. Jacobs and colleagues prospectively evaluated 67 TBI patients who had SPECT scan within four weeks of the initial injury and three months after the first scan. Of those, 33 patients showed no significant abnormalities on their initial SPECT scan and 97% experienced positive outcomes in their clinical symptoms within three months following TBI. They found 34 patients with pathologic initial SPECT scans, and 59% of these patients continued to encounter notable clinical symptoms at 3 months follow up. At 12 months following TBI, pathologic SPECT scans were found to have increased sensitivity up to 95% [151]. Romero and colleagues designed a study aiming to correlate cerebral perfusion changes in TBI patients with depressive symptoms. They reported that mild levels of depressive symptoms following mild TBI do not contribute to subacute cerebral hypoperfusion, as quantified measures of SPECT perfusion were unable to distinguish among mild TBI patients regarding the presence of depressive symptoms [152].

SPECT can detect brain activation changes during complex cognitive tasks in patients with chronic symptoms following TBI [153]. Hattori and colleagues observed a pattern of activation in TBI patients different from that in healthy control subjects. Patients with mild TBI presented with less activation in the cerebellum despite relatively more activation in the prefrontal cortex. They demonstrated functional frontocerebellar dissociation because of possibly impaired connection fibers due to axonal injury or degeneration after TBI. These findings were hypothesized to exist due to a mechanism of compensation [154]. Because of the diffuse nature of TBI, post-injury regional cerebral blood flow is not always proportionate with metabolism in SPECT, despite its good correlation in healthy individuals [155].

## 5. Neuromonitoring after TBI

During the last decades, multimodal neuromonitoring technology has made great progress. It has been shown to play an important role in treatment of TBI, preventing secondary brain injury and promoting better patient outcome.

### 5.1. Intracranial Pressure (ICP) Monitoring and Cerebral Autoregulation

Continuous intracranial pressure (ICP) monitoring is widely used clinically after severe TBI. Although clinical studies in ICP monitoring overall showed controversial results, recent trials indicate lower mortality and better outcome in patients with monitored and controlled ICP. The Brain Trauma Foundation recommends with level II evidence to treat ICP above 20 mmHg [156,157,158,159,160,161].

Two established invasive methods are the application of an intraparenchymal microsensor and the intraventricular catheter. The intraventricular catheter is considered the gold standard, since it measures global ICP and allows drainage of cerebrospinal fluid as treatment or administration of drugs. Intraparenchymal ICP monitors, on the other hand, are frequently used as a first-line monitoring method and are considered to be safe and of equal accuracy to intraventricular measurements [162,163,164]. Devices have been developed offering the ability to monitor ICP and parenchymal temperature. Treatment based on ICP monitoring alone may not be enough in the complex heterogenic entity of TBI. Several studies showed that treatment based on multimodal monitoring is superior to ICP alone. This induces investigation of further indices and factors with better correlation to mortality and outcome [157,165].

ICP is frequently used in TBI treatment to calculate the cerebral perfusion pressure (CPP) involving the patient’s mean arterial pressure. With level II and III evidence the Brain Trauma Foundation recommends to maintain a CPP threshold between 50 and 70 mmHg [160,162]. ICP pulse-wave-patterns have been a research topic focusing on computerized analysis of morphological changes of ICP waves. Recently an algorithm has been introduced as Morphological Cluster and Analysis of Intracranial Pressure (MOCAIP) that might offer a promising way to measure cerebral perfusion and detect real-time cerebral hypoperfusion [166].

The future in monitoring seeks for noninvasive methods with equal accuracy as invasive techniques. Neuroimaging, as described in the previous section, can be considered as noninvasive single point of time of ICP evaluation. Recent studies showed possible ICP estimation by measuring the optic sheath diameter using ultrasound [167] or magnetic resonance imaging [163,168,169].

Cerebral autoregulation stands for the regulation of the cerebral blood flow by dilation and constriction of cerebral arterioles in response to changes of cerebral perfusion pressure. In neurocritical patients a dysregulated autoregulation of pressure flow or reactivity has been linked to poor outcome. Developing the best correlating monitoring coefficient for tailored treatment and prognosis is a central research topic in TBI [170].

Currently known indices using further monitoring methods with promising results are shown in Table 4, involving data derived from additional methods like transcranial Doppler, NIRS or measurement of brain tissue oxygen.

**Table 4:** Indices of cerebral autoregulation.

Index	Reference	Title
PRx (pressure reactivity index)	Howells *et al*., 2014 [171]	An optimal frequency range for assessing the pressure reactivity index in patients with traumatic brain injury
L-PRx (low-frequency pressure reactivity index)	Depreitere *et al*., 2014 [172]	Pressure autoregulation monitoring and cerebral perfusion pressure target recommendation in patients with severe traumatic brain injury based on minute-by-minute monitoring data
Mx (mean index, TCD-derived)	Zweifel *et al*., 2008 [173]	Continuous monitoring of cerebrovascular pressure reactivity in patients with head injury
Sx (systolic flow index, TCD-derived)	Budohoski *et al*., 2012 [174]	Monitoring cerebral autoregulation after head injury. Which component of transcranial Doppler flow velocity is optimal
PAx (pressure-amplitude index, TCD-derived)	Radolovich *et al*., 2011 [175]	Pulsatile intracranial pressure and cerebral autoregulation after traumatic brain injury
PI (pulsatility index, TCD-derived)	Melo *et al*., 2011 [176]	Transcranial Doppler can predict intracranial hypertension in children with severe traumatic brain injuries
COx (cerebral oximetry index, NIRS-derived, Somanetics)	Brady *et al*., 2007 [177]	Continuous time-domain analysis of cerebrovascular autoregulation using NIRS
TOx (tissue oxygenation index, NIRS-derived, Hämamatsu)	Steiner *et al*., 2009 [178]	NIRS can monitor dynamic cerebral autoregulation in adults
HVx (haemoglobin volume index, NIRS-derived, Somanetics)	Lee *et al*., 2012 [179]	Noninvasive autoregulation monitoring in a swine model of pediatric cardiac arrest
THx (total haemoglobin reactivity index, IRS-derived, Hämamatsu)	Zweifel *et al*., 2010 [180]	Noninvasive monitoring of cerebrovascular reactivity with NIRS in head-injured patients
ORx (brain tissue oxygen reactivity index)	Lang *et al*., 2014 [181]	Changes in cerebral partial oxygen pressure and cerebrovascular reactivity during intracranial pressure plateau waves

Abbrev.: TCD = Transcranial Doppler.

### 5.2. Brain Oxygen Monitoring

In 2007, brain oxygen monitoring was added into the Brain Trauma Foundation guidelines with a level III evidence to treat brain tissue oxygen tension thresholds below 15 mmHg. In the past decade several studies showed a correlation between low brain oxygen levels and higher mortality rate and unfavorable outcome [182,183]. Combined monitoring of ICP and brain tissue oxygen tension has shown to be superior to ICP alone [184,185]. Additionally, brain oxygenation offers a new variable to evaluate cerebral autoregulation and cerebrovascular reactivity as mentioned before in Table 3. There are measuring devices with two different techniques to monitor the partial oxygen pressure in the surrounding brain tissue offering simultaneous monitoring of partial carbon dioxide pressure, temperature and ICP. The Licox microcatheter is the most frequently used in clinical settings and is based on the Clark principle, a polarographic technique. The Neurovent-PTO sondes are optical technique based, the same as the OxyLite catheter that is only used under experimental condition. Unfortunately values measured with different sensors cannot be compared directly [186]. In the management of patients with TBI the use of Licox has been demonstrated in several trials to improve outcome [184]. Neurovent has shown promising results in initial studies of clinical cases [187].

A non-invasive way to assess real-time regional cortical oxygenation is with NIRS (near-infrared spectroscopy). It is most frequently used in cardiovascular surgery to detect cerebral oxygenation changes during surgical cardiac arrest during cardiopulmonary bypass. NIRS is non-invasive, but has a number of limitations causing highly variable results and, therefore, is only used for restricted indications [183]. Track changes may be monitored with this method, and a baseline for comparison is needed. In case of TBI, brain tissue oxygenation may be already compromised from the beginning and an absolute measurement is necessary for evaluation [188]. Recently in a piglet model with controlled reduction of arterial blood pressure, NIRS has shown to be suitable for long-term monitoring of cerebral autoregulation and blood flow and high correlation to Laser Doppler Flowmetry [173]. The device, CerOx 3110, using NIRS technology, combined with ultrasound, has been introduced as a method based on ultrasound-tagged near-infrared spectroscopy (UT-NIRS) to measure regional cerebral tissue oxygenation and blood flow. Correlation to jugular bulb venous monitoring could be provided for the ipsilateral hemisphere, whereas correlation of Licox and CerOx oxygenation measures could not be shown in severe TBI [189].

The heterogeneous entity of TBI makes generalizable equations and overall studies difficult. Recently a new method for predictive analysis after TBI with symbolic regression modeling has been introduced. Symbolic regression was used as a computational framework to study the dynamic parameters of ICP, brain temperature and brain tissue oxygen to generate equations from real-time data. This may lead to development of dynamic smart monitoring devices that continuously collect data and redefine individual critical boundaries with constant improvement of accuracy [190].

### 5.3. Electrophysiologic Monitoring

Electrophysiology is a well-established non-invasive diagnostic method for neurological monitoring, prognostic and follow-up evaluation and also for neuromodulatory therapy after TBI. In experimental settings it has been broadly used in models of neurological diseases and in cerebral hypoxia and cardiac arrest models [191,192]. In animal models the monitoring of sensory evoked potentials was shown to be useful in detecting the course of brain injury and early phases of injury progression [193].

Especially in mild cases of TBI electrophysiology monitoring proved to be superior to standard clinical evaluations only in detecting cerebral dysfunction [194,195]. The analysis of event-related potentials during working memory tasks indicated decreased EEG-wave-amplitudes in patients with mild TBI compared to control group [196]. A direct correlation between ICP elevations and durations of EEG burst and suppression intervals could be shown. Short periods of electrical activity in EEG of burst-suppressed patients were followed by transient ICP elevations. It was suggested in context of neurovascular coupling after brain injury [197]. Cortical spreading depolarization is another research area as a possible pathophysiological mechanism after TBI. It has demonstrated to have high positive and negative predictive value for delayed cerebral ischemia after TBI [198].

It should be briefly mentioned at this point, that besides monitoring, EEG may play a role in treatment of brain injury. Young TBI-patients showed improvement in cognitive functions and concussion symptoms. Additionally, thalamo-cortical connection in follow-up MRI was increased after EEG neurofeedback therapy [199].

### 5.4. Microdialysis

Since the early 1990s, cerebral microdialysis has become an established neurochemical monitoring method to analyze low molecular weight biomarkers and to measure metabolic energy crisis or cellular distress in brain tissue after injury and ischemia [187,200,201]. The most frequently studied molecules are glucose, lactate, pyruvate, glycerol and glutamate (Table 5) [202].

**Table 5:** Clinically used microdialysis biomarkers [203].

Marker for	Biomarker	Clinical Significance
Energy metabolism	Glucose	↓ in hypoxia/ischemia
	Lactate	↑ in hypoxia/ischemia
	Pyruvate	↓ in hypoxia/ischemia
	Lactate/Pyruvate Ratio	↑ in hypoxia/ischemia = best marker for anaerobic metabolism
Cellular distress	Glycerol	↑ with destruction of cell membrane structure and generation of free radicals
	Glutamate	↑ in hypoxia/ischemia and excitotoxicity

Treatment of glucose levels measured by microdialysis is controversial. Available data demonstrate hyperglycemia is common in TBI patients and is related to poor outcome. However, current clinical trials have not proven any benefit of tight cerebral glucose controlled treatment. So far, monitoring of cerebral glucose has only proven to detect and predict infarction risk depending on the position of the microdialysis catheter [204]. Despite monitoring of brain tissue alone, microdialysis could be used to analyze cerebrospinal fluid (CSF) continuously. Microdialysis has demonstrated measurements of CSF-glucose and CSF-lactate comparable with conventional laboratory CSF analysis. Elevated glucose and lactate levels correlated with poor outcome, although the lactate-pyruvate-ratio has been unaffected [205].

One cornerstone in intracranial microdialysis research is focused on lactate and the lactate-pyruvate-ratio (LPR). Although lactate has been considered to be an excellent biomarker for oxygen deprivation, the mechanism of brain lactate metabolism and shuttling remains poorly understood in case of TBI [206]. Lactate uptake into the brain appears to be upregulated after TBI to potentially serve greater demands for energy substrate [207]. Beneficial effect of exogenous lactate infusion on cerebral energy metabolism after TBI is controversial and highly discussed [206,208]. Based on the lactate concentration, the ratio of lactate and pyruvate is a marker for anaerobic cerebral metabolism [209]. However, it must be interpreted in consideration of other parameters to distinguish between two types of LRP elevations. In cases of low oxygenation due to an ischemic event, LRP is elevated in process of anaerobic metabolism. The other type occurs under normal or even high oxygenation; LRP may be increased due to activated aerobic hyperglycolysis from deprivation of energetic substrates [210]. This phenomenon is called metabolic crisis and is common and deleterious after TBI, yet it has not been well studied so far [211]. The following Table 6 shows other promising, but less common biomarkers lacking further investigation for evidence of better utility.

**Table 6:** Potenial microdialysis biomarkers.

Biomarker	Significance	Reference
Serum albumin	BBB disruption	Blyth *et al*., 2009 [212]
Hemoglobin subunit α and ß	Red blood cell degradation	Babu *et al*., 2012 [213]
Serotransferrin	Free iron in the brain tissue	Park *et al*., 2011 [214]
Creatine kinase B-type	Enhancing predictive sensitivity of S100B as a biomarker	Bazarian *et al*., 2006 [215]
Cystatin C	Increased autophagy	Liu *et al*., 2013 [216]
Apolipoprotein A-1 and E	Membrane remodeling due to cellular trauma	Mahley *et al*., 2012 [217]
α-2-HS-glycoprotein (Fetuin-A)	Systemic response to cerebral injury	Wang and Sama *et al*., 2012 [218]
Complement C3	Activation of the innate immune response to injury	Ducruet *et al.*, 2009 [219]

In stroke models, disruption of ion homeostasis especially in the neurogliovascular unit has shown to be pathophysiologically involved in development of cerebral edema and, therefore, increased morbidity and mortality after ischemia [220,221,222].

In an experimental TBI model to explore the cerebral ionic profile, microdialysis probes were inserted into matrices and perfused with mock cerebrospinal fluid. With this method the actual ionic concentration in the extracellular fluid may be calculated based on the concentration in the microdialysate [223]. Elevated extracellular brain potassium was shown to be associated with neuronal damage in experimental models [224]. A recent pilot study after subarachnoid hemorrhage suggested it may serve as a biomarker for secondary brain injury [225].

Aiming for technical catheter improvement, a recent paper introduced a surface modified microdialysis catheter coated with PluronicF-127. This method have shown promising results in improving accuracy and precision of protein biomarker sampling in a porcine brain injury model [226].

Overall microdialysis is a promising method to offer sampling and measuring metabolic mechanisms, tissue biomarkers and pharmacokinetics in almost any human tissue and organ [227,228].

### 5.5. Brain-on-Chip

A new innovative technology to monitor changes at the level of axonal and neuronal networks has been recently introduced. The brain-on-a-chip device is an experimental strain injury model that maintains three dimensional cell architecture. It has been used to visualize hippocampal axonal response and biochemical changes induced by diffuse axonal injury after TBI. Changes in the mitochondrial membrane potentials have shown to lead to axonal degeneration [229,230].

## 6. Treatment Strategies and Modalities after TBI

### 6.1. Stem Cell Based Therapy

Stem cell-based therapy has been tested and has been well-documented for years in various disease models [231] and especially in stroke research [232]. In this review, we will not elaborate on the known mechanisms underlying the therapeutic effectiveness induced by stem cell administration, but stress some new concepts and mechanisms to show current updates in the field. Regenerative approaches include cell replacement, neurotrophic factor delivery, manipulation of intracellular signaling, bridging and artificial substrates, and modulation of the immune response [233].

6.1.1. “Biobridges” Paved by Stem Cells

Tajiri and colleagues proposed a theory of stem cell-paved formation of “biobridges” in experimental models of TBI. Rats subjected to TBI were intracerebrally transplanted with SB623 (gene-modified human mesenchymal stromal cells). One month after TBI, they observed notable increases in endogenous cellular proliferation as well as immature neural differentiation in the peri-injured cortical areas and subventricular zone, along with a stream of migrating cells along the corpus callosum of SB623 transplanted TBI rats. Furthermore, at 3 months post-TBI, they observed enhanced cellular proliferation and neural differentiation in peri-injured cortical areas of SB623 transplanted TBI animals, accompanied by a solid stream of neuronally-labeled cells migrating not only along but also across the corpus callosum from the subventricular zone to the impacted cortex. TBI rats, which had not received mesenchymal stromal cells, exhibited, elevated cellular proliferation; however the newly formed cells were “trapped” within the subventricular zone (SVZ) and cerebral cortex and did not migrate to the impacted cerebral cortex. Likewise, these animals showed a 2 and 9-fold upregulation of the matrix metalloproteinase-9 (MMP) activity and expression, respectively, at 1 and 3 months post-TBI. MMP-9 was upregulated in the TBI group, but reverted back to sham levels at 3 months post-TBI. To provide further evidence that the implanted stromal cells facilitated the formation of the biobridge, Tajiri and colleagues used primary rat cortical cells grown both alone and co-cultured with stromal cells either in the presence or absence of the MMP-9 inhibitor and revealed, by using migratory cell assay, enhanced migration of primary rat cortical cells with stromal cells, while these were suppressed by treatment with the MMP-9 inhibitor [234].

#### 6.1.2. Stabilized BBB by Stem Cells

Disruption of the blood brain barrier and vascular neuronal network following TBI often leads to uncontrolled inflammation and tissue damage [63,235]. Pati and colleagues designed a study to investigate the effects of mesenchymal stem cells (MSCs) on endothelial cells and vascular permeability using both *in vitro* and *in vivo* experimental methods. MSCs were cocultured with human umbilical vascular endothelial cells. At 2 and 24 h after injury MSCs was administered intravenously and rats were sacrificed 3 days after TBI. These results showed that MSCs exert a potent stabilizing effect on human vascular endothelial cells by increasing VE-cadherin expression and enhanced VE-cadherin/β-catenin interaction. Consistent with this, intravenous administration of MSCs increased VE-cadherin levels in brain homogenates and decreased TBI-associated enhanced BBB permeability. In addition, Pati and colleagues examined the interaction between tissue inhibitor of matrix metalloproteinase-3 (TIMP3) and mesenchymal stem cells due to regulation of blood brain barrier integrity 24 and 72 h post TBI. Results showed knockdown of TIMP3 in MSCs fully abrogates the protective effects of these stem cells on BBB permeability after TBI by reducing occludin-1 and VE-cadherin expression in the penumbra. In addition, following intravenous recombinant TIMP3 (rTIMP3) application after TBI, BBB integrity was preserved by increasing the expression of Occludin-1 and VE-Cadherin [236].

#### 6.1.3. Reduction of Oxidative Stress by Stem Cells

TBI consists of a primary insult resulting from a mechanical force inflicted on the head, and is amplified by a secondary insult that increases reactive oxygen species (ROS) production, excitatory amino acid release, energy consumption, and disturbed calcium homeostasis [237]. Li and colleagues have mentioned that in oxidative stress microvascular endothelial cells have an important role in redistribution and degradation of tight junction proteins [238]. Torrente and colleagues examined the role of hMSC-conditioned medium (hMSC-CM) in scratch-injured glioblastoma cell lines associated with metabolic impairment induced by glucose starvation [239]. An increase in wound closure in scratched cells treated with increasing hMSC-CM concentrations was observed with increased cell viability in glucose-deprived cells at 12 h following stem cell treatment. To assess the effect of 2% hMSC-CM in cell morphology, serial pictures were taken by the research group using a bright-field microscope and through measuring the distance from the injury site, they found that when cells are submitted to both scratch injury and glucose deprivation, hMSC-CM treatment increased cell polarity and revealed a higher capability of migration in cells toward injury. They proposed that migration and proliferation plays a role in wound regeneration and both processes are acting synergistically, and this effect is potentiated by stem cell medium. Due to the fact that increased reactive oxygen species production is a common secondary mechanism following brain injury, hMSC-CM administration was able to reduce them to basal levels, suggesting their ability to regulate oxidative stress [240].

#### 6.1.4. Immunomodulation by Stem Cells

Cellular injury following TBI is mostly related to an excessive inflammatory reaction near the injured area [241]. Galinda and colleagues reported a study of immunomodulatory properties of hMSC treatment [242] and results showed [243] downregulation of IL-6, and IL-10 mRNA levels in the acute setting but increased expression of IL-6 30 days after TBI. MSC transplantation into the injury site decreased the serum levels of IL-6, TNF-a, IL-4 and IL-10 in the acute phase of the model [244]. MSC secreted soluble factors capable of stimulating proliferation of NSC, *in vitro*, as well as increasing the expression of GFAP, a gene related to mature astrocytes, indicating that factors expressed by MSC could induce differentiation of NSC *in vivo* [245]. Zhang and colleagues designed a study to investigate the cerebral immunomodulatory effects of MSCs after TBI by systemic transplantation. They reported that injured cortex levels of proinflammatory cytokines IL-1β, IL-6, IL-17, TNF-α, IFN-γ and the chemokines MCP-1, MIP-2 after TBI were significantly decreased in the MSC-treatment group. Levels of the anti-inflammatory cytokines IL-10 and TGF-β1 were increased in the MSC-treatment group. There were no significant differences in levels of the cytokines IL-1α and IL-4. The immunomodulation occurred because mRNA levels of NF-κB were decreased from 12 to 48 h after MSC transplantation [246].

Bedii and colleagues performed an experiment to investigate the long-term effects of progenitor cells on microglia/macrophage phenotype and on the cognitive and motor functions of rodents after TBI. Each group was given two doses of its assigned multipotent adult progenitor cell therapy (MAPC) concentration at 2 and 24 h after controlled cortical impact (CCI) injury. They found improvements in average cognitive function as measured by Morris water maze (MWM) performance and motor function revealed by posture on the balance beam, and these were accompanied by localized reduction in numbers of activated microglia 120 days after injury. Both 24 to 120 h after TBI, MAPC treatment alters the ratio of proinflammatory microglia/macrophages to anti-inflammatory microglia/macrophages in dentate gyrus [247].

#### 6.1.5. White Matter Protection and Microvascular Integrity by Stem Cells

Park Katya and colleagues designed a study of white matter protection and microvascular integrity of endothelial progenitor cells (EPC) after TBI [248]. They examined coronal sections of the corpus callosum for evidence of β-APP accumulation after FPI and treatment. A significant decrease was found in the endothelial progenitor cell treatment group. Accumulation of β-APP in brain regions is detected following TBI. Axonal pathology after TBI is characterized by impaired axonal transport resulting in β-APP accumulation, axonal swelling, and ultimately axonal disconnection. In addition to white matter outcomes, EPC treatment was studied for possible improvements of microvascular outcomes in the corpus callosum. There was a significant difference in mean total capillary lengths in the corpus callosum between groups. EPC treatment resulted in improved microvascular lengths of individually measured capillary fragments. Because mechanical shearing of tissues and subsequent ischemia are major factors after TBI, they practiced a sub-lethal stretch injury model to isolate the mechanical injury component on cortical neuron cultures to determine if EPC-conditioned media was sufficient to rescue axon degeneration. However, no protective effect on axonal degeneration was found. To model the ischemic component of TBI, They performed oxygen–glucose deprivation (OGD) in primary neuron cultures and reported that OGD neuronal cultures treated with EPC-conditioned media had a significant decrease in the number of degenerating axons relative to OGD alone. In order to confirm that the protective effects were a result of the endothelial progenitor cells and not a result of nonspecific proliferating cell effects, fibroblast-conditioned media was added to a subset of cortical neuron cultures. No rescue of axon degeneration was found to occur in cortical neurons subjected to OGD and treated with fibroblast-conditioned media.

### 6.2. Nanotechnology Based Therapy

Due to current advances in nanotechnology, neuroscience research has initiated application of nanotechnology as novel bioengineering therapies for the treatment of nervous system injuries including TBI. Our discussion includes newly-developed nanomaterials instead of reviewing the well-studied nanomaterial like sealants, fusogens, chitosan and hydrophilic, nonionic polymers (poloxamers, poloxamines, and polyethylene glycol).

#### 6.2.1. Perfluorocarbons

PerfluoroCarbons are formed of carbon and fluorine and emulsions and can easily be dissolved in large quantities of gases, including O_2_ and CO_2_ [249]. Previous studies displayed their ability to reduce infarct volumes and improve neurological function in experimental cerebral ischemic models when administered intravenously [250]. Zhengwen and colleagues conducted a study on neuroprotective effects of a 3rd generation perfluorocarbon, called oxycyte, following TBI in rats. They concluded that injured animals treated with a lower or higher dose of oxycyte had significant improvement in cognitive function, and injured animals that received either dose of oxycyte had significantly less neuronal cell loss in the hippocampal CA3 region. A lower dose of oxycyte significantly improved mitochondrial oxygen consumption levels. The same authors previously demonstrated that perfluorocarbons combined with 100% O2 treatment can significantly increase cerebral oxygenation and enhance mitochondrial function after lateral fluid percussion injury in a rat model [251]. The use of perfluorocarbons in TBI could prove beneficial for reducing neuronal loss as well as mitochondrial functioning and decrease the neurodegenerative effects caused by the injury.

#### 6.2.2. Polyethylene Glycol-Functionalized Hydrophilic Carbon Clusters (PEG-HCCs)

PEG-HCCs are a class of biologically compatible carbon-based nanovectors which can be loaded with hydrophobic drugs, and when mixed with an antibody forms a noncovalent formulation capable of targeted drug delivery *in vitro* [252]. Marcano and colleagues conducted an *in vitro* study to evaluate the characteristics of PEG-HCCs designed specifically to address cerebrovascular dysfunction that accompanies TBI. The authors concluded that PEG-HCCs maintain antioxidant capacity sufficient for biological relevancy and targeting oxidatively stressed brain endothelial cells. When targeting the brain, they preserve their ability to alleviate oxidative stress in cultured brain endothelial cells [253].

Cell membrane repair mechanisms, advances to crossing the blood brain barrier, drug delivery to the injured brain, and nanostructure scaffolds for neural tissue engineering will be deterministic in the use of nanotechnology interventions as a prospective TBI treatment strategy.

### 6.3. Preconditioning in TBI Treatment

Prophylactic interventions involving methods of preconditioning (PC) may help individuals at high risk for brain injury or as a preoperative treatment for brain surgery, reducing the impact of primary and secondary brain damage [254]. For these reasons, translating basic science into chemical preconditioning allows clarification of the underlying mechanisms responsible for tissue tolerance and neuroprotection after cerebral injury, and may provide support for clinical applications.

#### 6.3.1. Hyperbaric Oxygen Treatment (HBOT)

Hyperbaric oxygen therapy (HBOT) has been defined as a neurotherapeutic method for brain repair, providing oxygen-enriched air to patients in a closed chamber pressurized above one atmosphere absolute (1ATA). The combination of hyperoxia and hyperbaric pressure leads to significant improvements in tissue oxygenation as well as mitochondrial metabolism with anti-apoptotic and anti-inflammatory effects [255,256,257,258,259,260]. HBO has proved to be neuroprotective against ischemic and hemorrhagic brain injury [261,262].

Wang and colleagues demonstrated that HBOT administered 2 days post injury resulted in significantly reduced overall neurological deficit scores and decreased neuronal apoptosis in brain tissues, and proposed the effective window for HBOT was 12 h post TBI [263]. Harch and colleagues tested 90-min sessions twice daily of HBOT at 1.5 ATA, starting 50 days after initial brain injury, and after 40 days of HBOT the MWM spatial learning performance in the HOBT groups improved significantly, and correlated with increased cerebrovascular density [263,264].

Harch and colleagues in 2010 reported dramatic improvement in a series of 15 patients treated with HBOT 1.5 in a clinical trial of military acquired TBI [265]. Multiple case reports show improvement in symptoms and functional brain scans for combat-related blast-related TBI [266,267]; hyperbaric oxygen-therapy (1.5 ATA) in treating sports related TBI/CTE was reported in two case reports [268]. A randomized clinical trial of the use of HBOT in persistent post-concussion symptoms (PCS) in military service members was released in 2015. Their results support that supplemental administration of breathing 100% oxygen at 1.5 ATA or air at 1.2 ATA for 60 min is well tolerated and improves symptoms and quality of life compared with local care management of those without chamber intervention. However, there were no observed differences between HBOT and sham group receiving air at 1.2 ATA. These outcomes suggest that the observed improvements were not oxygen mediated but may reflect nonspecific improvements related to placebo effects.

Discussions continue regarding the effectiveness of HBOT, but recent publications regarding the cognitive benefits of HBOT leave the medical community with an opportunity to pursue and conduct further research into its therapeutic benefits for the growing population of TBI.

#### 6.3.2. Glutaminergic Precoditioning

Glutamate (*N*-methyl-d-aspartate) is known as the main excitatory amino acid in the central nervous system and its activation is one of the mechanisms of brain damage after TBI. Glutamatergic excitotoxicity contributes to primary and secondary damage [269]. PC using glutamate has been suggested as a mechanism of neuroprotection when given in low doses [270,271]. Glutamate PC provides protection not only against cellular damage by inducing a state of cellular tolerance to a future lethal event [272,273], but also induces the trophic effects of glutamate on neuronal cell reorganization [274]. Though one study reports finding impairments with long term memory, preventing excitotoxicity related to increased calcium influx stimulated by NMDA receptors is of paramount importance in halting the cascade of damage that follows TBI [275].

Based on the current research and findings, the mechanisms of NMDA-mediated preconditioning (NMDA-PC) include increased excitability, induction of mild oxidative stress, modulation of bioenergetics, ionic homeostasis and the modulation of glutamatergic transmission within non-excitotoxic levels. Although the neural mechanisms involved in NMDA preconditioning are not completely understood, literature has shown that there exists a time–dependent approach of NMDA-PC and that it is maintained up to 48 h after administration [276], showing upregulation of proteins related to bioenergetics (e.g., creatine kinase) and processing (Heat Shock Protein 70). Furthermore, proteins involved in synaptic vesicle neurotransmitter accumulation have been downregulated. A study by Boeck and colleagues found that mitochondrial chain respiratory enzymes and creatine kinase may be associated with tolerance induction from previous NMDA preconditioning. TBI stimulation is decreased and ATP levels are maintained at a constant level by increasing the creatine kinase levels up to 24 after TBI [277].

Alterations in memory, cognitive, and mood impairments require a better understanding, and studies on the glutamatergic system are of paramount importance to clarify questions to reverse the pathologic cellular cascade underlying the progression of cell death. Prophylactic interventions involving PC may help individuals at high risk for brain injury or as a preoperative treatment for brain surgery [271]. For these reasons, translating basic science into chemical preconditioning allows clarification of the underlying mechanisms responsible for tissue tolerance and neuroprotection after cerebral injury, and may provide support for clinical applications.

### 6.4. Hormones in TBI Treatment

#### 6.4.1. Erythropoietin

Erythropoietin (EPO) is a 34-kDa glycoprotein hematopoietic growth factor belonging to the type I cytokine superfamily [278], with receptors on neurons, glia cells, astrocytes, and cerebral endothelial cells [279,280,281]. It performs CNS functions such as limiting the production of MMP-9 activity, cell death, reactive oxygen species [282] and glutamate, reversal of vasospasm, neurotransmission modulation, promotion of angiogenesis [283,284,285,286], anti-inflammatory actions [280,287] and anti-apoptotic [288,289] properties due to antagonism to pro-inflammatory cytokines such as tumor necrosis factor [290,291]. Recombinant human erythropoietin (rhEPO) plays protective roles in TBI including enhancing neurogenesis, restoring spatial memory [287], reducing neuronal apoptosis, down-regulating activation of glial cells and complement receptor type 3 [292], attenuating necrosis volume [293], modifying the endogenous indices of oxidative stress [294] and protecting the ultrastructure of pneumocyte type II cells [295]. It also improves functional outcomes via an increase in the mobilization of endothelial progenitor cells and in subsequent angiogenesis [296].

The effects of EPO on animal models proved to have positive effects on neuroprotection following TBI. EPO and analogs can reduce lesion volume and improve neurobehavioral outcomes in animal models of TBI. Due to recent clinical trials supporting the use of EPO associated with reduced mortality in trauma patients [297] and findings that link EPO-amplified neurogenesis with improved spatial learning following TBI, as well as attenuating necrosis volume, it has become an attractive treatment for TBI, reaching the main goal of neuroprotection: to prevent or decrease secondary damage and stimulate repair through enhancing adult endogenous neurogenesis.

Clinical trials on the efficacy of EPO treatment in patients with TBI yield conflicting results, and without rigorous, fine-tuned, and detailed preclinical evaluation, it is unlikely that novel neuroprotective drugs will prove effective when tested in large, time-consuming, and expensive human clinical trials. Because of its promising results in preclinical studies, it is important to improve the methodological quality of pre-clinical studies in order to efficiently progress towards EPO treatment in humans.

#### 6.4.2. Progesterone

Progesterone (PG) is a well-known hormone that functions primarily on the reproductive system. Recent decades have given new hope to its use besides its function as sex hormone. It has been found to act in several models of TBI, serving as a neuroprotector [298]. Thus, it gives justice to the name Baulieu coined, back in 1996, referring to it as a neurosteroid, as it is both synthesized *de novo* or from precursors in the brain [299]. This neuroprotective hormone is produced in the brain by neurons and glial cells, and the latter is the cell by which its neuroprotective actions are believed to derive. Cerebral edema, inflammation, apoptosis and oxidative stress include some of the pathophysiologic changes that occur after TBI. Not only acting as an anti-oxidant and upregulating GABA (neurotransmitter), PG seems to provide additional neuroprotection by reducing cerebral edema, and decreasing inflammation and apoptosis. Reducing inflammation is achieved through downregulating the expression of proinflammatory cytokines though microglia and astroglia. Cerebral edema is reduced, in part, by regulating the expression of aquaporin 4 (a water channel) present in astrocytes [298].

PG has been tried in clinical trials due to its pluripotential role after multiple animal studies supported its use in TBI. Phase II clinical trials have shown improvements in patients with TBI after treatment with PG including decreased mortality rate and increased functional independence scores when compared to placebo or healthy controls, respectively in moderate and severe TBI [300,301]. ProTECT started as a phase II clinical trial and established safety of the drug; thirty-day mortality in the treatment group was less than half that of the control group and moderately injured patients in the treatment group achieved better Glasgow Outcome Scale-Extended and Disability Rate Scale score than the placebo group [300].

In 2007, Wright and colleagues started ProTECT III, but found that after stratification of the patients based on severity of injury, no improvements in recovery were noted after giving progesterone or placebo to patients within four hours of injury, continuing treatment for 96 h, and the study was stopped [302]. Another recent Phase III clinical trial called SYNAPSE also concluded that PG had no benefit in severe TBI [303]. These randomized phase III clinical trials on severe TBI by Skolnick and colleagues in 2014 and Wright and colleagues in 2007, based on the premise that PG used in experimental animal studies shows reductions in cerebral edema along with anti-inflammatory and antiapoptotic effects that limit secondary injury. Through early administration of progesterone, these secondary injuries involved in TBI might be avoided. The SYNAPSE clinical trials showed no significant difference on the Glasgow Coma Scale (GCS) between the placebo and progesterone treatment given within 8 h of injury [302]. The outcomes of these trials require the medical community to rethink the standardized measures used to quantify TBI (GCS and Marshall classification) and move towards a more multidimensional approach for an individualized treatment [303].

In conjunction with this and other reports, the dynamics of TBI stretch beyond a monotherapeutic approach to target single receptors or specific mechanisms [304,305], and an approach that encompasses both the multiple direct and indirect injury mechanisms must be developed in order to provide effective treatment for TBI [303].

### 6.5. Antioxidants in TBI Treatment

Recently, the literature reveals more options in TBI treatment with the use of antioxidants in the clinical setting in the near future.

#### 6.5.1. U-83836E

The first antioxidant to mention is U-83836E a non-steroidal compound that functions as a lipopolysaccharide (LP) inhibitor and free radical destroyer. It not only decreases posttraumatic LP and preserves mitochondrial respiratory function, but also has calcium buffering capacities [306]. Its capacity to inhibit calpain-mediated cytoskeletal degradation in CCI TBI in mice has been recently acknowledged in the literature. This explains the detailed interaction between disruptions in neuronal Ca^2+^-homeostasis and posttraumatic LP and calpain mediated cytoskeletal damage [307]. These are inhibited by U-83836E, thereby preventing a cascade of secondary injury and allowing neuroprotection to take on a positive role in TBI.

#### 6.5.2. Melatonin

Melatonin has been well studied in the field of circadian rhythms and sleep; its functions in the brain also give it neuroprotectant qualities. It acts as both a direct scavenger of free radicals and an indirect regulator of endogenous enzyme expression [308]. Advantages of melatonin as a CNS injury therapeutic include its lipophilicity, brain penetrability, and low potential for side effects [309]. In TBI models, melatonin increased antioxidant levels in the brain, decreased NF-Kappaβ activation and improved cognitive function [310,311,312], though a combination of melatonin and minocycline in rat CCI model did not show benefits in cognitive function using melatonin alone or in combination.

#### 6.5.3. Tempol

Tempol, a stable membrane permeable nitroxide, has a 1 h to several days treatment window to achieve mitochondrial protection [313] and locomotor recovery respectively [314], and is also associated with the metabolism of many reactive oxygen species and reactive nitrogen species (ROS/RNS) in CCI-TBI model [315,316]. Tempol is able to reduce posttrauma LP and protein nitration-induced oxidative damage, allowing preservation of mitochondrial bioenergetics, and decrease calpain mediated cytoskeletal damage, thereby decreasing neurodegeneration [317]. Furthermore, Tempol decreased brain edema and improved neurological recovery in rat contusion head model.

#### 6.5.4. Resveratrol

Resveratrol, known to be present in high amounts in red wine and grapes, has since early 2000 emerged as a potential antioxidant with neuroprotective effects. Resveratrol shows promising abilities as a neuroprotective agent in TBI models, possibly inhibiting lipid peroxidation [318,319,320]. The use of resveratrol in an experimental model of TBI was able to counteract oxidative damage and prevent the depletion of the antioxidant glutathione and also resulted in a reduction of infarct area [321]. The use of antioxidants in sequestering ROS and other harmful molecules is a promising strategy to increase neuroprotection in TBI [318]. Part of the antioxidant effect of exogenous substances such as flavonoids may be due to the induction of endogenous antioxidants. Nrf2 activators may be prime candidates for the attenuation of oxidative stress and subsequent neurotoxicity induced by TBI [322].

### 6.6. Hypothermia

Since 1943, inducing hypothermia as a treatment in which body temperature is kept at 32–34 °C has been applied in various brain injuries aiming to decrease brain metabolism and reduce neuronal swelling [323]. Interventions achieving a more modest temperature reduction than induced hypothermia include drug therapies (e.g.,: paracetamol, acetaminophen, non-steroidal anti-inflammatory drugs [324]) and physical therapies (e.g.,: bedside fans, tepid sponging, ice packs, cooling blankets [325]). Studies in recent years used different temperature ranges in order to achieve good patient outcomes. Improving patient outcomes is also linked to prevention of cerebral hypoxia after TBI [326]. Induced hypothermia aims to fight against such detrimental effects [327,328], and positive results were achieved by reducing hypoxic events following TBI with the use of brain tissue oxygen–guided cerebral perfusion pressure management and therapeutic mild hypothermia (predefined as 33–35 °C) [329,330,331,332]. Hypothermic patients had lower values than normothermic individuals. A slow rewarming over 24 h after rapidly achieving the target therapy prevented acute worsening of intracranial hypertension in the first 72-h following TBI [333,334]. A study done by Takashi and colleagues [335] concluded that temperature control of 35–35.5 °C is sufficient to control intracranial hypertension without inducing cardiac dysfunction and oxygen debt [336]. Thus, hypothermia may reduce intracerebral pressure earlier than a 72-h window and decrease TBI induced inflammation.

Even though the mechanisms responsible for the actions of hypothermia in the treatment of TBI remain to be elucidated, mild induced hypothermia seems to have an association with the expression connexin-43 and glutamate-transporter-1 in the hippocampus following TBI in rats. Mild hypothermia improved TBI induced brain edema and neurological functional deficits, and it is accompanied by down regulation in expression of connexin-43, normally increased after TBI, and upregulation of glutamate-transporter-1 levels that are normally decreased following TBI [337]. In accordance with previous studies, a model of fluid percussion injury, post-traumatic hypothermia significantly attenuated cell death within the hippocampus and attenuated caspase-3 upregulation, thereby reducing markers of apoptosis [338].

A review by Cochrane and colleagues concluded that there is no evidence that induced hypothermia is beneficial in the treatment of traumatic head injury [339]. In addition, it suggested that there is an increase in pulmonary infections with the intervention of hypothermia compared to controls [340]. While considering the numerous variables in different clinical trials, the Brain Trauma Foundation states that six clinical tries of moderate quality did not clearly associate hypothermia with consistent and statistically significant reductions in mortality, but did find benefit in the fact that the same patients were more likely to have favorable neurological outcomes, defined as GCS scores of 4 or 5. In addition, it suggested that hypothermia may reduce mortality if cooling is maintained for more than 48 h [341].

There are two large ongoing trials of long-term hypothermia for TBI. One is the Eurotherm 3235 trial in Europe [323]. Hypothermia of 32–35 °C will be continued for at least 48 h until ICP is stabilized at normal level and then the patients will be re-warmed at a rate of 1 °C/4 h. The other is the POLAR trial for severe TBI patients. In this study, within 3 h of injury the patient will be rapidly cooled down and then maintained at 33 °C for 3 days [342]. Rewarming will occur at a rate of 0.17 °C/h and will be titrated to intracranial pressure (ICP) control. Results of this clinical trials aim to clarify questions remaining in regards to the role of hypothermia in TBI. Challenges still remain, and a multicenter randomized trial needs to be undertaken in order to clear the debate in regards to the mechanisms of hypothermia in relation to TBI. Further investigations into hypothermia could provide information on the benefits and protocols for optimal use not only in each age bracket, but also according to intensity of injury.

### 6.7. Inhibitor Interventions for TBI Treatment

#### 6.7.1. CR8

CR8 is a CDK (cyclin-dependent kinase) inhibitor and second-generation analog with promising therapeutic potential [343]. It has been used in research using the lateral fluid percussion injury (LFP) model of TBI and enables DNA synthesis in neurons, microglia/macrophages and astrocytes to occur [344] after 7 and 14 days post TBI. The involvement of cell cycle activators (CCA) in the pathological mechanisms of neuronal cell death with the CNS has been reported [345,346,347,348,349,350]. After LFP injury, CR8 downregulates the expression of cell cycle markers such as CDK1, n-myc phosphorylation [349], and other cell cycle proteins [350,351,352]. Neuroprotection is achieved by reducing CCA upregulation in neurons through the inhibition of CDKs through CR8. On neurobehavior tests, CR8 improved memory retention and reduced LFP-induced impairment in fear-based contextual and emotional memory function in the passive avoidance task [353]. CDK inhibition significantly improves hippocampal neuronal survival [348,349,350]. Furthermore, CR8 preserves neurons in the hippocampus and surrounding areas, resulting in cognitive improvements. Signs of depression normally present in LFP injured rats were greatly reduced with treatment of CR8. At the cellular level, CR8 decreases microglia and astrocyte activation, thereby preventing further damage to the CNS.

Cell cycle activators and their role in increased neurodegeneration and chronic neuroinflammation now face a strong opponent. CR8 is able to fight the pathophysiology of TBI, and combat the S-phase induction of cells that happens with CCA induced TBI as seen in the LFP model [353]. In conclusion, selective CDK inhibitors bring promise to treatment options for TBI in the clinical setting through its multi-faceted neuroprotective capacities associated with inhibition of CCA-dependent neurodegeneration and neural cell associated inflammation.

#### 6.7.2. Statins

Statins are 3-hydroxy-3-methylglutaryl coenzyme A (HMGA) reductase inhibitors, and not only act to reduce cholesterol, but influence both the acute and chronic phases of TBI [354,355,356,357,358,359,360]. A series of effects can be achieved through the use of statins on brain edema, BBB integrity and brain microvascular endothelial cells [361], cerebral blood flow, neuroinflammation, axonal injury, cell death, and trophic factor production. This is made possible by the influence statins have on several molecular outcomes such as TUNEL staining, CREB, Akt, eNOS, FOXO1, NF-kB, GSK3, cytokines, BrdU labeling, blood vessel formation, and vascular endothelial growth factor and increased expression and secretion of tissue inhibitor of metalloproteinase-1 and 2 [357,360].

Studies show improvements in behavior and histology with atorvastatin and simvastatin. Atorvastatin has a longer half-life and active metabolites for greater protection against neuronal death than simvastatin [362]. Despite this, Lu and colleagues reported simvastatin promoted less hippocampal CA3 cell death and improved MWM performance after CCI when compared to atorvastatin [363]. In concordance with Lu and colleagues other studies have shown simvastatin to better penetrate BBB. Wang and colleagues found in 2007 that atorvastatin and simvastatin decreased functional neurological deficits after TBI in mice, with improvements in short-term and long-term functional performance through the preservation of hippocampal neurons seen on histology, and inhibition of inflammatory cytokine MRNA expression in brain parenchyma. In addition, statins not only improved cerebral hemodynamics after TBI, but also contributed to improvements involving the decreased cerebral perfusion state that happens following TBI in pretreatment conditions [362].

According to studies done by Ramirez and colleagues 2011 simvastatin is the most effective statin. It has the power to decrease inflammation [364], increase cerebral diameter of vessels around the lesion site, and increase proliferation of endothelial cells in the neurogenic regions while decreasing neuronal loss in non neurogenic regions of hippocampal CA3 regions after TBI [365]. Simvastatin and atorvastatin improve behavioral outcomes, reduce hippocampal degeneration, and improve cerebral blood flow after experimental traumatic brain injury [362].

Plausible explanations for neurologic recovery lies in the understanding that simvastatin may enhance proliferation and neurogenesis as shown in cultured neural cells, and is able to increase the expression of notch-1 proteins, demonstrated in chronic simvastatin treatment [366]. In addition, neural cell division in the dentate gyrus and subventricular zone guarantees regeneration of neurons in the CNS [367]. Simvastatin induced both astrocytic and neuronal differentiation during *in vitro* experiments, but quantitative data shows that simvastatin significantly increased neuron populations. In addition, previous studies have shown that simvastatin inhibits the activation of microglial cells and astrocytes after TBI [360].

Currently, atorvastatin is undergoing a phase II clinical trial with a 7-day regimen in order to help fill in the gaps regarding efficacy of treatment in the months following TBI. Though the effects of chronic administration of statins are still unknown in the literature, its ability to decrease neuronal cell loss and control inflammation are favorable and offer a novel therapeutic strategy for acute TBI after closed head injury.

#### 6.7.3. Acetylcholinesterase Inhibitors

Neuroinflammation is one of the major causes for increased neuropathology and neurobehavioral changes after single or repeated exposures to blast activity [368,369,370,371,372,373]. Recent studies on alterations of acetylcholinesterase activity in specific areas of the brain in mice after repeated blasts prompted researchers to evaluate gene expression related to cholinergic and inflammatory pathways [374]. Significant down-regulation of cholinergic receptors including muscarinic 2 and nicotinic alpha 7 occurred in the midbrain of mice exposed to repeated blasts. Muscarinic and nicotinic alpha 7 acetylcholine receptors are essential elements of the anti-inflammatory pathways and down-regulation of these receptors in the midbrain region after blast induces the pro-inflammatory pathways leading to neuropathology and neurobehavioral deficits [375,376,377]. Concurrently, GABA and glutamate receptors in the midbrain are down-regulated after repeated blast exposure. Pro-inflammatory cytokines and chemokines are released both centrally and peripherally after blast exposure [369,370,378]. Furthermore, significant up-regulation of multiple interleukins, tumor necrosis factor, and their receptors during cDNA microarray analysis of various brain regions of repeated blast exposed mice showed confirmed the presence and initiation of pro inflammatory pathways. The pro-inflammatory marker for myeloperoxidase expression was significantly higher in the cerebellum of blast exposed mice [347]. Currently, there are no clinical trials exploring the uses of ACE inhibitors after TBI.

### 6.8. Lithium Used for TBI Treatment

Neuroprotective in nature, and acting across multiple pathways, lithium serves as a potential therapeutic agent for treatment of neurological deficits and damage, as well as neuronal protection and functional recovery after TBI [379]. Patients with TBI often suffer from depression, a disease found to reduce brain levels of BDNF [380,381], but lithium is able to increase BDNF levels in the brain [381,382,383,384]. Lithium increases nerve growth factor (NGF) in the rat frontal cortex, hippocampus, amygdala, and forebrain [382,383,385,386,387], as well as vascular endothelial growth factor (VEGF), throttling an increase in the number of astrocytes, oligodendroglia, neurons, and decreasing the size of the lesion thereby improving functional outcomes [388,389,390]. Lithium prevents stress-induced reductions in VEGF levels, thereby stimulating neurogenesis and angiogenesis. In preclinical studies, both chronic [391,392] and short-term uses of lithium have been found to activate neurogenesis [393]. In addition, therapeutic levels of lithium stimulate proliferation of nestin-positive progenitor cells in brain neurons [394], as proliferation of adult hippocampal progenitor cells involving Wnt/beta-catenin activation occurred after GSK-3-beta-inhibition [395]. TNF-alpha directly disturbs BBB integrity, leading to secondary injuries including cerebral edema and leukocyte infiltration [396]. A study performed investigating the effects of etanercept and lithium chloride found decreased TNF-alpha activity with subsequent decrease in cerebral damage after administration of the therapeutic agents [396].

TBI often leads to not only acute injury but also long-term neurodegeneration, and is one of the major risk factors for developing Alzheimer’s disease (AD). A strong indication of AD or neurodegeneration includes the presence of Beta amyloid (AB) peptide deposits, and these deposits increase after TBI in animal models and in patients with head trauma [396]. Because of lithium’s ability to inhibit GSK-3 activity, it can reduce beta amyloid peptide deposits [397]. In a study on an animal model of TBI, mice were treated with lithium after TBI and there was a reduction in beta amyloid load through B-secretase inhibition. BACE1 is the major B-secretase involved in production of beta amyloid in neurons [398], and BACE1 levels increase after TBI in both human and rat models [399,400,401]. The use of lithium after TBI blocked BACE1 expression, thereby reduced the AB load. This, in part, contributed to improved spatial learning and memory with increased hippocampal preservation as determined by improved neurobehavioral testing compared to TBI and sham groups [397].

Regulation of histone deacetylase (HDAC) inhibition regulation is neuroprotective in nature through its ability to restore histone acetylation levels and correct transcriptional deficits in various models of brain disorders, specifically TBI rodent models [402,403,404,405]. A recent study in 2013 by Fengshan and colleagues indicated that the effects of combined subtherapeutic valproate (VPA)–another mood stabilizing drug–and lithium in a mouse model of TBI [406]. Both lithium and VPA maintain the BBB through inhibition of both HDAC and TBI-induced MMP-9 overexpression [407,408]. The combined treatment of sub-effective doses of both lithium and VPA significantly reduced lesion volume while preserving BBB integrity three days post TBI. This was not observed in the groups with lithium or VPA alone. However, lithium alone showed improvements in fine motor neurobehavior at days 14 and 21, while combined treatment had more profound effects with significantly fewer Fluoro-Jade B (FJB)–a dye used to label degenerating neurons-positive cells at days 7, 14, and 21 [406]. The advantage of subtherapeutic levels of both drugs causes less side effects, improves tolerance to long term use, and targets the different cell pathways involved in the complex pathophysiology of TBI [409].

## 7. Conclusions

TBI research literature is published every year in great quantity and animal models of TBI are essential to clarify underlying injury mechanisms, to assess the safety and efficacy of novel therapies prior to clinical trials [410] and to better understand the injury mechanism that drive its progression [411]. This indicates the urgency to explore optimal combinations of preventive and therapeutic measurements for TBI. In this review, we stress updates in TBI tracing, monitoring and treatment while updating the historical progress in these areas. The past decades witnessed tremendous advances in all those areas described in this review.

TBI is a complex and heterogeneous disease that requires personalized tracing, monitoring and treatment in clinical practice according to the severity, course, age, gender and co-morbidities associated with injury. An individual specific therapeutic option would benefit TBI victims to achieve the ideal structural restoration preserving lives and decreasing not only costs but also long term comorbidities associated with this condition.

## References

[1] Hayes R.L., Jenkins L.W., Lyeth B.G. (1992). Neurotransmitter-mediated mechanisms of traumatic brain injury: Acetylcholine and excitatory amino acids. J. Neurotrauma.

[2] Faden A.I. (1996). Pharmacologic treatment of acute traumatic brain injury. JAMA.

[3] McIntosh T.K., Saatman K.E., Raghupathi R., Graham D.I., Smith D.H., Lee V.M., Trojanowski J.Q. (1998). The dorothy russell memorial lecture. The molecular and cellular sequelae of experimental traumatic brain injury: Pathogenetic mechanisms. Neuropathol. Appl. Neurobiol..

[4] McIntosh T.K., Juhler M., Wieloch T. (1998). Novel pharmacologic strategies in the treatment of experimental traumatic brain injury: 1998. J. Neurotrauma.

[5] Kermer P., Klöcker N., Bähr M. (1999). Neuronal death after brain injury. Models, mechanisms, and therapeutic strategies *in vivo*. Cell Tissue Res..

[6] Graham D.I., McIntosh T.K., Maxwell W.L., Nicoll J.A. (2000). Recent advances in neurotrauma. J. Neuropathol. Exp. Neurol..

[7] Weaver S.M., Chau A., Portelli J.N., Grafman J. (2012). Genetic polymorphisms influence recovery from traumatic brain injury. Neuroscientist.

[8] Dardiotis E., Grigoriadis S., Hadjigeorgiou G.M. (2012). Genetic factors influencing outcome from neurotrauma. Curr. Opin. Psychiatry.

[9] Dardiotis E., Fountas K.N., Dardioti M., Xiromerisiou G., Kapsalaki E., Tasiou A., Hadjigeorgiou G.M. (2010). Genetic association studies in patients with traumatic brain injury. Neurosurg. Focus.

[10] Michael D.B., Byers D.M., Irwin L.N. (2005). Gene expression following traumatic brain injury in humans: Analysis by microarray. J. Clin. Neurosci..

[11] Gallek M.J., Ritter L. (2011). Central nervous system genomics. Annu. Rev. Nurs. Res..

[12] Conley Y.P., Alexander S. (2011). Genomic, transcriptomic, and epigenomic approaches to recovery after acquired brain injury. PMR.

[13] Di Pietro V., Amin D., Pernagallo S., Lazzarino G., Tavazzi B., Vagnozzi R., Pringle A., Belli A. (2010). Transcriptomics of traumatic brain injury: Gene expression and molecular pathways of different grades of insult in a rat organotypic hippocampal culture model. J. Neurotrauma.

[14] Fahlenkamp A.V., Coburn M., Czaplik M., Ryang Y.-M., Kipp M., Rossaint R., Beyer C. (2011). Expression analysis of the early chemokine response 4 h after *in vitro* traumatic brain injury. Inflamm. Res. Off. J. Eur. Histamine Res. Soc..

[15] White T.E., Ford G.D., Surles-Zeigler M.C., Gates A.S., Laplaca M.C., Ford B.D. (2013). Gene expression patterns following unilateral traumatic brain injury reveals a local pro-inflammatory and remote anti-inflammatory response. BMC Genomics.

[16] Hu Z., Yu D., Almeida-Suhett C., Tu K., Marini A.M., Eiden L., Braga M.F., Zhu J., Li Z. (2012). Expression of miRNAs and their cooperative regulation of the pathophysiology in traumatic brain injury. PLoS ONE.

[17] Lei P., Li Y., Chen X., Yang S., Zhang J. (2009). Microarray based analysis of microRNA expression in rat cerebral cortex after traumatic brain injury. Brain Res..

[18] Bhalala O.G., Srikanth M., Kessler J.A. (2013). The emerging roles of microRNAs in CNS injuries. Nat. Rev. Neurol..

[19] Redell J.B., Liu Y., Dash P.K. (2009). Traumatic brain injury alters expression of hippocampal microRNAs: Potential regulators of multiple pathophysiological processes. J. Neurosci. Res..

[20] Redell J.B., Zhao J., Dash P.K. (2011). Altered expression of miRNA-21 and its targets in the hippocampus after traumatic brain injury. J. Neurosci. Res..

[21] Liu N.-K., Xu X.-M. (2011). MicroRNA in central nervous system trauma and degenerative disorders. Physiol. Genomics.

[22] Weber M., Baker M.B., Moore J.P., Searles C.D. (2010). MiR-21 is induced in endothelial cells by shear stress and modulates apoptosis and eNOS activity. Biochem. Biophys. Res. Commun..

[23] Saugstad J.A. (2010). MicroRNAs as effectors of brain function with roles in ischemia and injury, neuroprotection, and neurodegeneration. J. Cereb. Blood Flow Metab..

[24] Redell J.B., Moore A.N., Ward N.H., Hergenroeder G.W., Dash P.K. (2010). Human traumatic brain injury alters plasma microRNA levels. J. Neurotrauma.

[25] Faul M., Xu L., Wald M., Coronado V. Traumatic Brain Injury in the United States: Emergency Department Visits, Hospitalizations and Deaths 2002–2006. http://www.cdc.gov/traumaticbraininjury/pdf/blue_book.pdf.

[26] Mondello S., Schmid K., Berger R.P., Kobeissy F., Italiano D., Jeromin A., Hayes R.L., Tortella F.C., Buki A. (2014). The challenge of mild traumatic brain injury: Role of biochemical markers in diagnosis of brain damage. Med. Res. Rev..

[27] Brophy G.M., Pineda J.A., Papa L., Lewis S.B., Valadka A.B., Hannay H.J., Heaton S.C., Demery J.A., Liu M.C., Tepas J.J. (2009). αII-Spectrin breakdown product cerebrospinal fluid exposure metrics suggest differences in cellular injury mechanisms after severe traumatic brain injury. J. Neurotrauma.

[28] Yokobori S., Hosein K., Burks S., Sharma I., Gajavelli S., Bullock R. (2013). Biomarkers for the clinical differential diagnosis in traumatic brain injury—a systematic review. CNS Neurosci. Ther..

[29] Balakathiresan N., Bhomia M., Chandran R., Chavko M., McCarron R.M., Maheshwari R.K. (2012). MicroRNA let-7i is a promising serum biomarker for blast-induced traumatic brain injury. J. Neurotrauma.

[30] Geyer C., Ulrich A., Gräfe G., Stach B., Till H. (2009). Diagnostic value of S100B and neuron-specific enolase in mild pediatric traumatic brain injury. J. Neurosurg. Pediatr..

[31] Marion D., Bullock M.R. (2009). Current and future role of therapeutic hypothermia. J. Neurotrauma.

[32] Dietrich W.D., Atkins C.M., Bramlett H.M. (2009). Protection in animal models of brain and spinal cord injury with mild to moderate hypothermia. J. Neurotrauma.

[33] Choi H.A., Badjatia N., Mayer S.A. (2012). Hypothermia for acute brain injury—mechanisms and practical aspects. Nat. Rev. Neurol..

[34] Truettner J.S., Alonso O.F., Bramlett H.M., Dietrich W.D. (2011). Therapeutic hypothermia alters microRNA responses to traumatic brain injury in rats. J. Cereb. Blood Flow Metab..

[35] Diamond B., Honig G., Mader S., Brimberg L., Volpe B.T. (2013). Brain-reactive antibodies and disease. Annu. Rev. Immunol..

[36] Zhang Z., Zoltewicz J.S., Mondello S., Newsom K.J., Yang Z., Yang B., Kobeissy F., Guingab J., Glushakova O., Robicsek S. (2014). W. Human traumatic brain injury induces autoantibody response against glial fibrillary acidic protein and its breakdown products. PLoS ONE.

[37] Ngankam L., Kazantseva N.V., Gerasimova M.M. Immunological markers of severity and outcome of traumatic brain injury. http://www.mediasphera.ru/journals/korsakov/776/eng/12266/.

[38] Goryunova A.V., Bazarnaya N.A., Sorokina E.G., Semenova N.Y., Globa O.V., Semenova Z.B., Pinelis V.G., Roshal L.M., Maslova O.I. (2007). Glutamate receptor autoantibody concentrations in children with chronic post-traumatic headache. Neurosci. Behav. Physiol..

[39] Neher M.D., Keene C.N., Rich M.C., Moore H.B., Stahel P.F. (2014). Serum biomarkers for traumatic brain injury. South. Med. J..

[40] Mondello S., Muller U., Jeromin A., Streeter J., Hayes R.L., Wang K.K.W. (2011). Blood-based diagnostics of traumatic brain injuries. Expert Rev. Mol. Diagn..

[41] Ghonemi M.O., Rabah A.A., Saber H.M., Radwan W. (2013). Role of phosphorylated neurofilament H as a diagnostic and prognostic marker in traumatic brain injury. Egypt. J. Crit. Care Med..

[42] Topolovec-Vranic J., Pollmann-Mudryj M.-A., Ouchterlony D., Klein D., Spence J., Romaschin A., Rhind S., Tien H.C., Baker A.J. (2011). The value of serum biomarkers in prediction models of outcome after mild traumatic brain injury. J. Trauma.

[43] Mondello S., Robicsek S.A., Gabrielli A., Brophy G.M., Papa L., Tepas J., Robertson C., Buki A., Scharf D., Jixiang M. (2010). αII-spectrin breakdown products (SBDPs): Diagnosis and outcome in severe traumatic brain injury patients. J. Neurotrauma.

[44] Papa L., Lewis L.M., Silvestri S., Falk J.L., Giordano P., Brophy G.M., Demery J.A., Liu M.C., Mo J., Akinyi L. (2012). Serum levels of ubiquitin C-terminal hydrolase distinguish mild traumatic brain injury from trauma controls and are elevated in mild and moderate traumatic brain injury patients with intracranial lesions and neurosurgical intervention. J. Trauma Acute Care Surg..

[45] Kobeissy F.H., Ottens A.K., Zhang Z., Liu M.C., Denslow N.D., Dave J.R., Tortella F.C., Hayes R.L., Wang K.K.W. (2006). Novel differential neuroproteomics analysis of traumatic brain injury in rats. Mol. Cell. Proteomics.

[46] Berger R.P., Adelson P.D., Pierce M.C., Dulani T., Cassidy L.D., Kochanek P.M. (2005). Serum neuron-specific enolase, S100B, and myelin basic protein concentrations after inflicted and noninflicted traumatic brain injury in children. J. Neurosurg..

[47] Rodríguez-Rodríguez A., Egea-Guerrero J.J., León-Justel A., Gordillo-Escobar E., Revuelto-Rey J., Vilches-Arenas A., Carrillo-Vico A., Domínguez-Roldán J.M., Murillo-Cabezas F., Guerrero J.M. (2012). Role of S100B protein in urine and serum as an early predictor of mortality after severe traumatic brain injury in adults. Clin. Chim. Acta.

[48] Vos P.E., Jacobs B., Andriessen T.M.J.C., Lamers K.J.B., Borm G.F., Beems T., Edwards M., Rosmalen C.F., Vissers J.L.M. (2010). GFAP and S100B are biomarkers of traumatic brain injury: An observational cohort study. Neurology.

[49] Okonkwo D.O., Yue J.K., Puccio A.M., Panczykowski D.M., Inoue T., McMahon P.J., Sorani M.D., Yuh E.L., Lingsma H.F., Maas A.I.R. (2013). Transforming research and clinical knowledge in traumatic brain injury (TRACK-TBI) investigators GFAP-BDP as an acute diagnostic marker in traumatic brain injury: Results from the prospective transforming research and clinical knowledge in traumatic brain injury study. J. Neurotrauma.

[50] Chittiboina P., Ganta V., Monceaux C.P., Scott L.K., Nanda A., Alexander J.S. (2013). Angiopoietins as promising biomarkers and potential therapeutic targets in brain injury. Pathophysiology.

[51] Chou S.H.-Y., Robertson C.S. (2014). Biomarkers of cellular injury and death in acute brain injury. Neurocrit. Care.

[52] Diaz-Arrastia R., Wang K.K.W., Papa L., Sorani M.D., Yue J.K., Puccio A.M., McMahon P.J., Inoue T., Yuh E.L., Lingsma H.F. (2014). Acute biomarkers of traumatic brain injury: Relationship between plasma levels of ubiquitin C-terminal hydrolase-L1 and glial fibrillary acidic protein. J. Neurotrauma.

[53] Czeiter E., Mondello S., Kovacs N., Sandor J., Gabrielli A., Schmid K., Tortella F., Wang K.K.W., Hayes R.L., Barzo P. (2012). Brain injury biomarkers may improve the predictive power of the IMPACT outcome calculator. J. Neurotrauma.

[54] Chabok S.Y., Moghadam A.D., Saneei Z., Amlashi F.G., Leili E.K., Amiri Z.M. (2012). Neuron-specific enolase and S100BB as outcome predictors in severe diffuse axonal injury. J. Trauma Acute Care Surg..

[55] Feala J.D., Abdulhameed M.D.M., Yu C., Dutta B., Yu X., Schmid K., Dave J., Tortella F., Reifman J. (2013). Systems biology approaches for discovering biomarkers for traumatic brain injury. J. Neurotrauma.

[56] Mechtler L.L., Shastri K.K., Crutchfield K.E. (2014). Advanced neuroimaging of mild traumatic brain injury. Neurol. Clin..

[57] Toyama Y., Kobayashi T., Nishiyama Y., Satoh K., Ohkawa M., Seki K. (2005). CT for acute stage of closed head injury. Radiat. Med..

[58] Gallagher C.N., Hutchinson P.J., Pickard J.D. (2007). Neuroimaging in trauma. Curr. Opin. Neurol..

[59] Pettus E.H., Povlishock J.T. (1996). Characterization of a distinct set of intra-axonal ultrastructural changes associated with traumatically induced alteration in axolemmal permeability. Brain Res..

[60] Hunter J.V., Wilde E.A., Tong K.A., Holshouser B.A. (2012). Emerging imaging tools for use with traumatic brain injury research. J. Neurotrauma.

[61] Wintermark M., Sanelli P.C., Anzai Y., Tsiouris A.J., Whitlow C.T. (2015). Imaging evidence and recommendations for traumatic brain injury: Advanced neuro- and neurovascular imaging techniques. Am. J. Neuroradiol..

[62] Metting Z., Rödiger L.A., de Keyser J., van der Naalt J. (2007). Structural and functional neuroimaging in mild-to-moderate head injury. Lancet Neurol..

[63] Glushakova O.Y., Johnson D., Hayes R.L. (2014). Delayed increases in microvascular pathology after experimental traumatic brain injury are associated with prolonged inflammation, blood-brain barrier disruption, and progressive white matter damage. J. Neurotrauma.

[64] Wu G., Xi G., Hua Y., Sagher O. (2010). T2* magnetic resonance imaging sequences reflect brain tissue iron deposition following intracerebral hemorrhage. Transl. Stroke Res..

[65] Geurts B.H.J., Andriessen T.M.J.C., Goraj B.M., Vos P.E. (2012). The reliability of magnetic resonance imaging in traumatic brain injury lesion detection. Brain Inj..

[66] Wang X., Wei X.-E., Li M.-H., Li W.-B., Zhou Y.-J., Zhang B., Li Y.-H. (2014). Microbleeds on susceptibility-weighted MRI in depressive and non-depressive patients after mild traumatic brain injury. Neurol. Sci..

[67] Tong K.A., Ashwal S., Holshouser B.A., Shutter L.A., Herigault G., Haacke E.M., Kido D.K. (2003). Hemorrhagic shearing lesions in children and adolescents with posttraumatic diffuse axonal injury: Improved detection and initial results. Radiology.

[68] Tong K.A., Ashwal S., Holshouser B.A., Nickerson J.P., Wall C.J., Shutter L.A., Osterdock R.J., Haacke E.M., Kido D. (2004). Diffuse axonal injury in children: Clinical correlation with hemorrhagic lesions. Ann. Neurol..

[69] Ryan N.P., Catroppa C., Cooper J.M., Beare R., Ditchfield M., Coleman L., Silk T., Crossley L., Beauchamp M.H., Anderson V.A. (2014). The emergence of age-dependent social cognitive deficits after generalized insult to the developing brain: A longitudinal prospective analysis using susceptibility-weighted imaging. Hum. Brain Mapp..

[70] Hinds T., Shalaby-Rana E., Jackson A.M., Khademian Z. (2015). Aspects of abuse: Abusive head trauma. Curr. Probl. Pediatr. Adolesc. Health Care.

[71] Geddes J.F., Vowles G.H., Hackshaw A.K., Nickols C.D., Scott I.S., Whitwell H.L. (2001). Neuropathology of inflicted head injury in children. II. Microscopic brain injury in infants. Brain J. Neurol..

[72] Gleckman A.M., Bell M.D., Evans R.J., Smith T.W. (1999). Diffuse axonal injury in infants with nonaccidental craniocerebral trauma: Enhanced detection by beta-amyloid precursor protein immunohistochemical staining. Arch. Pathol. Lab. Med..

[73] Niwa T., Aida N., Shishikura A., Fujita K., Inoue T. (2008). Susceptibility-Weighted Imaging Findings of Cortical Laminar Necrosis in Pediatric Patients. Am. J. Neuroradiol..

[74] Biousse V., Suh D.Y., Newman N.J., Davis P.C., Mapstone T., Lambert S.R. (2002). Diffusion-weighted magnetic resonance imaging in shaken baby syndrome. Am. J. Ophthalmol..

[75] Li N., Wang W.-T., Sati P., Pham D.L., Butman J.A. (2014). Quantitative assessment of susceptibility-weighted imaging processing methods. J. Magn. Reson. Imaging.

[76] Liu J., Kou Z., Tian Y. (2014). Diffuse axonal injury after traumatic cerebral microbleeds: An evaluation of imaging techniques. Neural Regen. Res..

[77] Edlow B.L., Wu O. (2012). Advanced neuroimaging in traumatic brain injury. Semin. Neurol..

[78] Fisher M., Bråtane B.T. (2012). Imaging of Experimental Stroke Models. Transl. Stroke Res..

[79] Goetz P., Blamire A., Rajagopalan B., Cadoux-Hudson T., Young D., Styles P. (2004). Increase in apparent diffusion coefficient in normal appearing white matter following human traumatic brain injury correlates with injury severity. J. Neurotrauma.

[80] Schlaug G., Siewert B., Benfield A., Edelman R.R., Warach S. (1997). Time course of the apparent diffusion coefficient (ADC) abnormality in human stroke. Neurology.

[81] Sevick R.J., Kanda F., Mintorovitch J., Arieff A.I., Kucharczyk J., Tsuruda J.S., Norman D., Moseley M.E. (1992). Cytotoxic brain edema: Assessment with diffusion-weighted MR imaging. Radiology.

[82] Barzó P., Marmarou A., Fatouros P., Hayasaki K., Corwin F. (1997). Contribution of vasogenic and cellular edema to traumatic brain swelling measured by diffusion-weighted imaging. J. Neurosurg..

[83] Hou D.J., Tong K.A., Ashwal S., Oyoyo U., Joo E., Shutter L., Obenaus A. (2007). Diffusion-weighted magnetic resonance imaging improves outcome prediction in adult traumatic brain injury. J. Neurotrauma.

[84] Moen K.G., Håberg A.K., Skandsen T., Finnanger T.G., Vik A. (2014). A longitudinal magnetic resonance imaging study of the apparent diffusion coefficient values in corpus callosum during the first year after traumatic brain injury. J. Neurotrauma.

[85] Galloway N.R., Tong K.A., Ashwal S., Oyoyo U., Obenaus A. (2008). Diffusion-weighted imaging improves outcome prediction in pediatric traumatic brain injury. J. Neurotrauma.

[86] Hudak A.M., Peng L., Marquez de la Plata C., Thottakara J., Moore C., Harper C., McColl R., Babcock E., Diaz-Arrastia R. (2014). Cytotoxic and vasogenic cerebral edemaedema in traumatic brain injury: Assessment with FLAIR and DWI imaging. Brain Inj..

[87] Moen K.G., Brezova V., Skandsen T., Håberg A.K., Folvik M., Vik A. (2014). Traumatic axonal injury: The prognostic value of lesion load in corpus callosum, brain stem, and thalamus in different magnetic resonance imaging sequences. J. Neurotrauma.

[88] Brezova V., Moen K.G., Skandsen T., Vik A., Brewer J.B., Salvesen O., Håberg A.K. (2014). Prospective longitudinal MRI study of brain volumes and diffusion changes during the first year after moderate to severe traumatic brain injury. NeuroImage Clin..

[89] Ito J., Marmarou A., Barzó P., Fatouros P., Corwin F. (1996). Characterization of edema by diffusion-weighted imaging in experimental traumatic brain injury. J. Neurosurg..

[90] Wei X.-E., Zhang Y.-Z., Li Y.-H., Li M.-H., Li W.-B. (2012). Dynamics of rabbit brain edema in focal lesion and perilesion area after traumatic brain injury: A MRI study. J. Neurotrauma.

[91] Huisman T.A.G.M., Sorensen A.G., Hergan K., Gonzalez R.G., Schaefer P.W. (2003). Diffusion-weighted imaging for the evaluation of diffuse axonal injury in closed head injury. J. Comput. Assist. Tomogr..

[92] Fiehler J., Knab R., Reichenbach J.R., Fitzek C., Weiller C., Röther J. (2001). Apparent diffusion coefficient decreases and magnetic resonance imaging perfusion parameters are associated in ischemic tissue of acute stroke patients. J. Cereb. Blood Flow Metab..

[93] Håberg A.K., Olsen A., Moen K.G., Schirmer-Mikalsen K., Visser E., Finnanger T.G., Evensen K.A.I., Skandsen T., Vik A., Eikenes L. (2014). White matter microstructure in chronic moderate-to-severe traumatic brain injury: Impact of acute-phase injury-related variables and associations with outcome measures. J. Neurosci. Res..

[94] Marquez de la Plata C., Ardelean A., Koovakkattu D., Srinivasan P., Miller A., Phuong V., Harper C., Moore C., Whittemore A., Madden C. (2007). Magnetic resonance imaging of diffuse axonal injury: Quantitative assessment of white matter lesion volume. J. Neurotrauma.

[95] Maegele M., Stuermer E.K., Hoeffgen A., Uhlenkueken U., Mautes A., Schaefer N., Lippert-Gruener M., Schaefer U., Hoehn M. (2015). Multimodal MR imaging of acute and subacute experimental traumatic brain injury: Time course and correlation with cerebral energy metabolites. Acta Radiol. Short Rep..

[96] Colgan N.C., Gilchrist M.D., Curran K.M. (2010). Applying DTI white matter orientations to finite element head models to examine diffuse TBI under high rotational accelerations. Prog. Biophys. Mol. Biol..

[97] Basser P.J., Pajevic S., Pierpaoli C., Duda J., Aldroubi A. (2000). *In vivo* fiber tractography using DT-MRI data. Magn. Reson. Med..

[98] Niogi S.N., Mukherjee P., Ghajar J., Johnson C., Kolster R.A., Sarkar R., Lee H., Meeker M., Zimmerman R.D., Manley G.T. (2008). Extent of microstructural white matter injury in postconcussive syndrome correlates with impaired cognitive reaction time: A 3T diffusion tensor imaging study of mild traumatic brain injury. Am. J. Neuroradiol..

[99] Jorge R.E., Acion L., White T., Tordesillas-Gutierrez D., Pierson R., Crespo-Facorro B., Magnotta V.A. (2012). White matter abnormalities in veterans with mild traumatic brain injury. Am. J. Psychiatry.

[100] Ptak T., Sheridan R.L., Rhea J.T., Gervasini A.A., Yun J.H., Curran M.A., Borszuk P., Petrovick L., Novelline R.A. (2003). Cerebral fractional anisotropy score in trauma patients: A new indicator of white matter injury after trauma. Am. J. Roentgenol..

[101] Rutgers D.R., Fillard P., Paradot G., Tadié M., Lasjaunias P., Ducreux D. (2008). Diffusion tensor imaging characteristics of the corpus callosum in mild, moderate, and severe traumatic brain injury. Am. J. Neuroradiol..

[102] Niogi S.N., Mukherjee P., McCandliss B.D. (2007). Diffusion tensor imaging segmentation of white matter structures using a Reproducible Objective Quantification Scheme (ROQS). NeuroImage.

[103] Gardner A., Kay-Lambkin F., Stanwell P., Donnelly J., Williams W.H., Hiles A., Schofield P., Levi C., Jones D.K. (2012). A systematic review of diffusion tensor imaging findings in sports-related concussion. J. Neurotrauma.

[104] Elbin R.J., Covassin T., Henry L., Whalen D.J., Wedge J., Kontos A.P. (2013). Sport-related concussion: “How many is too many?”. Transl. Stroke Res..

[105] Yuh E.L., Cooper S.R., Mukherjee P., Yue J.K., Lingsma H.F., Gordon W.A., Valadka A.B., Okonkwo D.O., Schnyer D.M., Vassar M.J. (2014). Diffusion tensor imaging for outcome prediction in mild traumatic brain injury: A TRACK-TBI study. J. Neurotrauma.

[106] Roberts R.M., Mathias J.L., Rose S.E. (2014). Diffusion Tensor Imaging (DTI) findings following pediatric non-penetrating TBI: A meta-analysis. Dev. Neuropsychol..

[107] Wozniak J.R., Krach L., Ward E., Mueller B.A., Muetzel R., Schnoebelen S., Kiragu A., Lim K.O. (2007). Neurocognitive and neuroimaging correlates of pediatric traumatic brain injury: A diffusion tensor imaging (DTI) study. Arch. Clin. Neuropsychol..

[108] Sidaros A., Engberg A.W., Sidaros K., Liptrot M.G., Herning M., Petersen P., Paulson O.B., Jernigan T.L., Rostrup E. (2008). Diffusion tensor imaging during recovery from severe traumatic brain injury and relation to clinical outcome: A longitudinal study. Brain J. Neurol..

[109] Messé A., Caplain S., Paradot G., Garrigue D., Mineo J.-F., Soto Ares G., Ducreux D., Vignaud F., Rozec G., Desal H. (2011). Diffusion tensor imaging and white matter lesions at the subacute stage in mild traumatic brain injury with persistent neurobehavioral impairment. Hum. Brain Mapp..

[110] Dodd A.B., Epstein K., Ling J.M., Mayer A.R. (2014). Diffusion Tensor Imaging Findings in Semi-Acute Mild Traumatic Brain Injury. J. Neurotrauma.

[111] Cubon V.A., Putukian M., Boyer C., Dettwiler A. (2011). A diffusion tensor imaging study on the white matter skeleton in individuals with sports-related concussion. J. Neurotrauma.

[112] Davenport N.D., Lim K.O., Armstrong M.T., Sponheim S.R. (2012). Diffuse and spatially variable white matter disruptions are associated with blast-related mild traumatic brain injury. NeuroImage.

[113] Fernandez-Miranda J.C., Engh J.A., Pathak S.K., Madhok R., Boada F.E., Schneider W., Kassam A.B. (2010). High-definition fiber tracking guidance for intraparenchymal endoscopic port surgery. J. Neurosurg..

[114] Sotak C.H. (2002). The role of diffusion tensor imaging in the evaluation of ischemic brain injury—A review. NMR Biomed..

[115] Shin S.S., Verstynen T., Pathak S., Jarbo K., Hricik A.J., Maserati M., Beers S.R., Puccio A.M., Boada F.E., Okonkwo D.O. (2012). High-definition fiber tracking for assessment of neurological deficit in a case of traumatic brain injury: Finding, visualizing, and interpreting small sites of damage. J. Neurosurg..

[116] Buxton R.B. (2013). The physics of functional magnetic resonance imaging (fMRI). Rep. Prog. Phys..

[117] Dijkhuizen R.M., van der Marel K., Otte W.M., Hoff E.I., van der Zijden J.P., van der Toorn A., van Meer M.P.A. (2012). Functional MRI and Diffusion Tensor Imaging of Brain Reorganization after Experimental Stroke. Transl. Stroke Res..

[118] Stevens M.C., Lovejoy D., Kim J., Oakes H., Kureshi I., Witt S.T. (2012). Multiple resting state network functional connectivity abnormalities in mild traumatic brain injury. Brain Imaging Behav..

[119] Kasahara M., Menon D.K., Salmond C.H., Outtrim J.G., Tavares J.V.T., Carpenter T.A., Pickard J.D., Sahakian B.J., Stamatakis E.A. (2011). Traumatic brain injury alters the functional brain network mediating working memory. Brain Inj..

[120] Palacios E.M., Sala-Llonch R., Junque C., Roig T., Tormos J.M., Bargallo N., Vendrell P. (2013). Resting-state functional magnetic resonance imaging activity and connectivity and cognitive outcome in traumatic brain injury. JAMA Neurol..

[121] Wilde E.A., Newsome M.R., Bigler E.D., Pertab J., Merkley T.L., Hanten G., Scheibel R.S., Li X., Chu Z., Yallampalli R. (2011). Brain imaging correlates of verbal working memory in children following traumatic brain injury. Int. J. Psychophysiol..

[122] Edlow B.L., Giacino J.T., Wu O. (2013). Functional MRI and outcome in traumatic coma. Curr. Neurol. Neurosci. Rep..

[123] Smits M., Dippel D.W.J., Houston G.C., Wielopolski P.A., Koudstaal P.J., Hunink M.G.M., van der Lugt A. (2009). Postconcussion syndrome after minor head injury: Brain activation of working memory and attention. Hum. Brain Mapp..

[124] Talavage T.M., Nauman E.A., Breedlove E.L., Yoruk U., Dye A.E., Morigaki K.E., Feuer H., Leverenz L.J. (2014). Functionally-detected cognitive impairment in high school football players without clinically-diagnosed concussion. J. Neurotrauma.

[125] Venkatesan U.M., Dennis N.A., Hillary F.G. (2015). Chronology and chronicity of altered resting-state functional connectivity after traumatic brain injury. J. Neurotrauma.

[126] Damoiseaux J.S., Rombouts S.A.R.B., Barkhof F., Scheltens P., Stam C.J., Smith S.M., Beckmann C.F. (2006). Consistent resting-state networks across healthy subjects. Proc. Natl. Acad. Sci. USA.

[127] Bonnelle V., Leech R., Kinnunen K.M., Ham T.E., Beckmann C.F., de Boissezon X., Greenwood R.J., Sharp D.J. (2011). Default mode network connectivity predicts sustained attention deficits after traumatic brain injury. J. Neurosci..

[128] Nathan D.E., Oakes T.R., Yeh P.H., French L.M., Harper J.F., Liu W., Wolfowitz R.D., Wang B.Q., Graner J.L., Riedy G. (2015). Exploring variations in functional connectivity of the resting state default mode network in mild traumatic brain injury. Brain Connect..

[129] Schomer D.L., Silva F.L. (2012). Da Niedermeyer’s Electroencephalography: Basic Principles, Clinical Applications, and Related Fields.

[130] Hari R., Salmelin R. (2012). Magnetoencephalography: From SQUIDs to neuroscience. NeuroImage.

[131] Huang M.-X., Nichols S., Baker D.G., Robb A., Angeles A., Yurgil K.A., Drake A., Levy M., Song T., McLay R. (2014). Single-subject-based whole-brain MEG slow-wave imaging approach for detecting abnormality in patients with mild traumatic brain injury. NeuroImage Clin..

[132] Lee A.K.C., Larson E., Maddox R.K. (2012). Mapping cortical dynamics using simultaneous MEG/EEG and anatomically-constrained minimum-norm estimates: An auditory attention example. J. Vis. Exp..

[133] Iwasaki M., Nakasato N., Shamoto H., Yoshimoto T. (2003). Focal magnetoencephalographic spikes in the superior temporal plane undetected by scalp EEG. J. Clin. Neurosci..

[134] Iwasaki M., Pestana E., Burgess R.C., Lüders H.O., Shamoto H., Nakasato N. (2005). Detection of epileptiform activity by human interpreters: Blinded comparison between electroencephalography and magnetoencephalography. Epilepsia.

[135] Huang M.-X., Huang C.W., Robb A., Angeles A., Nichols S.L., Baker D.G., Song T., Harrington D.L., Theilmann R.J., Srinivasan R. (2014). MEG source imaging method using fast L1 minimum-norm and its applications to signals with brain noise and human resting-state source amplitude images. NeuroImage.

[136] Tarapore P.E., Findlay A.M., Lahue S.C., Lee H., Honma S.M., Mizuiri D., Luks T.L., Manley G.T., Nagarajan S.S., Mukherjee P. (2013). Resting state magnetoencephalography functional connectivity in traumatic brain injury. J. Neurosurg..

[137] Blackband S.J. (1996). MR spectroscopy: Clinical applications and techniques. Radiography.

[138] Lin A.P., Liao H.J., Merugumala S.K., Prabhu S.P., Meehan W.P., Ross B.D. (2012). Metabolic imaging of mild traumatic brain injury. Brain Imaging Behav..

[139] Kumar A., Loane D.J. (2012). Neuroinflammation after traumatic brain injury: Opportunities for therapeutic intervention. Brain. Behav. Immun..

[140] Harris J.L., Yeh H.-W., Choi I.-Y., Lee P., Berman N.E., Swerdlow R.H., Craciunas S.C., Brooks W.M. (2012). Altered neurochemical profile after traumatic brain injury: 1H-MRS biomarkers of pathological mechanisms. J. Cereb. Blood Flow Metab..

[141] Henry L.C., Tremblay S., Boulanger Y., Ellemberg D., Lassonde M. (2010). Neurometabolic changes in the acute phase after sports concussions correlate with symptom severity. J. Neurotrauma.

[142] Vagnozzi R., Signoretti S., Tavazzi B., Floris R., Ludovici A., Marziali S., Tarascio G., Amorini A.M., di Pietro V., Delfini R. (2008). Temporal window of metabolic brain vulnerability to concussion: A pilot 1H-magnetic resonance spectroscopic study in concussed athletes—part III. Neurosurgery.

[143] Maudsley A.A., Govind V., Levin B., Saigal G., Harris L., Sheriff S. (2015). Distributions of magnetic resonance diffusion and spectroscopy measures with traumatic brain injury. J. Neurotrauma.

[144] Tollard E., Galanaud D., Perlbarg V., Sanchez-Pena P., Le Fur Y., Abdennour L., Cozzone P., Lehericy S., Chiras J., Puybasset L. (2009). Experience of diffusion tensor imaging and 1H spectroscopy for outcome prediction in severe traumatic brain injury: Preliminary results. Crit. Care Med..

[145] Nobili F., Frisoni G.B., Portet F., Verhey F., Rodriguez G., Caroli A., Touchon J., Calvini P., Morbelli S., de Carli F. (2008). Brain SPECT in subtypes of mild cognitive impairment. Findings from the DESCRIPA multicenter study. J. Neurol..

[146] Ichise M., Chung D.G., Wang P., Wortzman G., Gray B.G., Franks W. (1994). Technetium-99m-HMPAO SPECT, CT and MRI in the evaluation of patients with chronic traumatic brain injury: A correlation with neuropsychological performance. J. Nucl. Med. Off. Publ. Soc. Nucl. Med..

[147] Amen D.G., Newberg A., Thatcher R., Jin Y., Wu J., Keator D., Willeumier K. (2011). Impact of playing American professional football on long-term brain function. J. Neuropsychiatry Clin. Neurosci..

[148] Haacke E.M., Duhaime A.C., Gean A.D., Riedy G., Wintermark M., Mukherjee P., Brody D.L., DeGraba T., Duncan T.D., Elovic E. (2010). Common data elements in radiologic imaging of traumatic brain injury. J. Magn. Reson. Imaging.

[149] Kant R., Smith-Seemiller L., Isaac G., Duffy J. (1997). Tc-HMPAO SPECT in persistent post-concussion syndrome after mild head injury: Comparison with MRI/CT. Brain Inj..

[150] Newberg A.B., Serruya M., Gepty A., Intenzo C., Lewis T., Amen D., Russell D.S., Wintering N. (2014). Clinical comparison of 99mTc exametazime and 123I Ioflupane SPECT in patients with chronic mild traumatic brain injury. PLoS ONE.

[151] Jacobs A., Put E., Ingels M., Bossuyt A. (1994). Prospective evaluation of technetium-99m-HMPAO SPECT in mild and moderate traumatic brain injury. J. Nucl. Med. Off. Publ. Soc. Nucl. Med..

[152] Romero K., Black S.E., Feinstein A. (2014). Differences in cerebral perfusion deficits in mild traumatic brain injury and depression using single-photon emission computed tomography. Front. Neurol..

[153] Raji C.A., Tarzwell R., Pavel D., Schneider H., Uszler M., Thornton J., van Lierop M., Cohen P., Amen D.G., Henderson T. (2014). Clinical Utility of SPECT Neuroimaging in the Diagnosis and Treatment of Traumatic Brain Injury: A Systematic Review. PLoS ONE.

[154] Hattori N., Swan M., Stobbe G.A., Uomoto J.M., Minoshima S., Djang D., Krishnananthan R., Lewis D.H. (2009). Differential SPECT activation patterns associated with PASAT performance may indicate frontocerebellar functional dissociation in chronic mild traumatic brain injury. J. Nucl. Med. Off. Publ. Soc. Nucl. Med..

[155] Abu-Judeh H.H., Singh M., Masdeu J.C., Abdel-Dayem H.M. (1998). Discordance between FDG uptake and technetium-99m-HMPAO brain perfusion in acute traumatic brain injury. J. Nucl. Med. Off. Publ. Soc. Nucl. Med..

[156] Yuan Q., Wu X., Sun Y., Yu J., Li Z., Du Z., Mao Y., Zhou L., Hu J. (2015). Impact of intracranial pressure monitoring on mortality in patients with traumatic brain injury: A systematic review and meta-analysis. J. Neurosurg..

[157] Le Roux P. (2014). Intracranial pressure after the BEST TRIP trial: A call for more monitoring. Curr. Opin. Crit. Care.

[158] Talving P., Karamanos E., Teixeira P.G., Skiada D., Lam L., Belzberg H., Inaba K., Demetriades D. (2013). Intracranial pressure monitoring in severe head injury: Compliance with Brain Trauma Foundation guidelines and effect on outcomes: A prospective study. J. Neurosurg..

[159] Alali A.S., Fowler R.A., Mainprize T.G., Scales D.C., Kiss A., de Mestral C., Ray J.G., Nathens A.B. (2013). Intracranial pressure monitoring in severe traumatic brain injury: Results from the American College of Surgeons Trauma Quality Improvement Program. J. Neurotrauma.

[160] Prabhakar H., Sandhu K., Bhagat H., Durga P., Chawla R. (2014). Current concepts of optimal cerebral perfusion pressure in traumatic brain injury. J. Anaesthesiol. Clin. Pharmacol..

[161] Colton K., Yang S., Hu P.F., Chen H.H., Stansbury L.G., Scalea T.M., Stein D.M. (2014). Responsiveness to therapy for increased intracranial pressure in traumatic brain injury is associated with neurological outcome. Injury.

[162] Kirkman M.A., Smith M. (2014). Intracranial pressure monitoring, cerebral perfusion pressure estimation, and ICP/CPP-guided therapy: A standard of care or optional extra after brain injury?. Br. J. Anaesth..

[163] Kristiansson H., Nissborg E., Bartek J., Andresen M., Reinstrup P., Romner B. (2013). Measuring elevated intracranial pressure through noninvasive methods: A review of the literature. J. Neurosurg. Anesthesiol..

[164] Koskinen L.-O.D., Olivecrona M. (2005). Clinical experience with the intraparenchymal intracranial pressure monitoring Codman MicroSensor system. Neurosurgery.

[165] Melhem S., Shutter L., Kaynar A. (2014). A trial of intracranial pressure monitoring in traumatic brain injury. Crit. Care Lond. Engl..

[166] Hu X., Glenn T., Scalzo F., Bergsneider M., Sarkiss C., Martin N., Vespa P. (2010). Intracranial pressure pulse morphological features improved detection of decreased cerebral blood flow. Physiol. Meas..

[167] Soldatos T., Chatzimichail K., Papathanasiou M., Gouliamos A. (2009). Optic nerve sonography: A new window for the non-invasive evaluation of intracranial pressure in brain injury. Emerg. Med. J. EMJ.

[168] Geeraerts T. (2013). Noninvasive surrogates of intracranial pressure: Another piece added with magnetic resonance imaging of the cerebrospinal fluid thickness surrounding the optic nerve. Crit. Care Lond. Engl..

[169] An H., Sen S., Chen Y., Powers W.J., Lin W. (2012). Noninvasive measurements of cerebral blood flow, oxygen extraction fraction, and oxygen metabolic index in human with inhalation of air and carbogen using magnetic resonance imaging. Transl. Stroke Res..

[170] Hawthorne C., Piper I. (2014). Monitoring of intracranial pressure in patients with traumatic brain injury. Front. Neurol..

[171] Howells T., Johnson U., McKelvey T., Enblad P. (2015). An optimal frequency range for assessing the pressure reactivity index in patients with traumatic brain injury. J. Clin. Monit. Comput..

[172] Depreitere B., Güiza F., van den Berghe G., Schuhmann M.U., Maier G., Piper I., Meyfroidt G. (2014). Pressure autoregulation monitoring and cerebral perfusion pressure target recommendation in patients with severe traumatic brain injury based on minute-by-minute monitoring data. J. Neurosurg..

[173] Zweifel C., Lavinio A., Steiner L.A., Radolovich D., Smielewski P., Timofeev I., Hiler M., Balestreri M., Kirkpatrick P.J., Pickard J.D. (2008). Continuous monitoring of cerebrovascular pressure reactivity in patients with head injury. Neurosurg. Focus.

[174] Budohoski K.P., Reinhard M., Aries M.J.H., Czosnyka Z., Smielewski P., Pickard J.D., Kirkpatrick P.J., Czosnyka M. (2012). Monitoring cerebral autoregulation after head injury. Which component of transcranial Doppler flow velocity is optimal?. Neurocrit. Care.

[175] Radolovich D.K., Aries M.J.H., Castellani G., Corona A., Lavinio A., Smielewski P., Pickard J.D., Czosnyka M. (2011). Pulsatile intracranial pressure and cerebral autoregulation after traumatic brain injury. Neurocrit. Care.

[176] Melo J.R.T., Rocco F.D., Blanot S., Cuttaree H., Sainte-Rose C., Oliveira-Filho J., Zerah M., Meyer P.G. (2011). Transcranial Doppler can predict intracranial hypertension in children with severe traumatic brain injuries. Childs Nerv. Syst..

[177] Brady K.M., Lee J.K., Kibler K.K., Smielewski P., Czosnyka M., Easley R.B., Koehler R.C., Shaffner D.H. (2007). Continuous time-domain analysis of cerebrovascular autoregulation using near-infrared spectroscopy. Stroke J. Cereb. Circ..

[178] Steiner L.A., Pfister D., Strebel S.P., Radolovich D., Smielewski P., Czosnyka M. (2009). Near-infrared spectroscopy can monitor dynamic cerebral autoregulation in adults. Neurocrit. Care.

[179] Lee J.K., Yang Z.-J., Wang B., Larson A.C., Jamrogowicz J.L., Kulikowicz E., Kibler K.K., Mytar J.O., Carter E.L., Burman H.T. (2012). Noninvasive autoregulation monitoring in a swine model of pediatric cardiac arrest. Anesth. Analg..

[180] Zweifel C., Castellani G., Czosnyka M., Helmy A., Manktelow A., Carrera E., Brady K.M., Hutchinson P.J.A., Menon D.K., Pickard J.D. (2010). Noninvasive monitoring of cerebrovascular reactivity with near infrared spectroscopy in head-injured patients. J. Neurotrauma.

[181] Lang E.W., Kasprowicz M., Smielewski P., Pickard J., Czosnyka M. (2014). Changes in cerebral partial oxygen pressure and cerebrovascular reactivity during intracranial pressure plateau waves. Neurocrit. Care.

[182] Marini C.P., Stoller C., Shah O., Policastro A., Lombardo G., Asensio J.A., Hu Y.C., Stiefel M.F. (2014). The impact of early flow and brain oxygen crisis on the outcome of patients with severe traumatic brain injury. Am. J. Surg..

[183] Oddo M., Bösel J. (2014). Monitoring of brain and systemic oxygenation in neurocritical care patients. Neurocrit. Care.

[184] Keddie S., Rohman L. (2012). Reviewing the reliability, effectiveness and applications of Licox in traumatic brain injury. Nurs. Crit. Care.

[185] Le Roux P.D., Oddo M. (2013). Parenchymal brain oxygen monitoring in the neurocritical care unit. Neurosurg. Clin. N. Am..

[186] Beynon C., Kiening K.L., Orakcioglu B., Unterberg A.W., Sakowitz O.W. (2012). Brain tissue oxygen monitoring and hyperoxic treatment in patients with traumatic brain injury. J. Neurotrauma.

[187] Purins K., Lewén A., Hillered L., Howells T., Enblad P. (2014). Brain tissue oxygenation and cerebral metabolic patterns in focal and diffuse traumatic brain injury. Front. Neurol..

[188] Scheeren T.W.L., Schober P., Schwarte L.A. (2012). Monitoring tissue oxygenation by near infrared spectroscopy (NIRS): Background and current applications. J. Clin. Monit. Comput..

[189] Rosenthal G., Furmanov A., Itshayek E., Shoshan Y., Singh V. (2014). Assessment of a noninvasive cerebral oxygenation monitor in patients with severe traumatic brain injury. J. Neurosurg..

[190] Narotam P.K., Morrison J.F., Schmidt M.D., Nathoo N. (2014). Physiological complexity of acute traumatic brain injury in patients treated with a brain oxygen protocol: Utility of symbolic regression in predictive modeling of a dynamical system. J. Neurotrauma.

[191] Jia X., Koenig M.A., Shin H.-C., Zhen G., Pardo C.A., Hanley D.F., Thakor N.V., Geocadin R.G. (2008). Improving neurological outcomes post-cardiac arrest in a rat model: Immediate hypothermia and quantitative EEG monitoring. Resuscitation.

[192] Kang X., Xiong W., Koenig M., Puttgen H.A., Jia X., Geocadin R., Thakor N. (2009). Long-term assessment of post-cardiac-arrest neurological outcomes with somatosensory evoked potential in rats. Eng. Med. Biol. Soc..

[193] Ordek G., Proddutur A., Santhakumar V., Pfister B.J., Sahin M. (2014). Electrophysiological monitoring of injury progression in the rat cerebellar cortex. Front. Syst. Neurosci..

[194] Gosselin N., Bottari C., Chen J.-K., Petrides M., Tinawi S., de Guise É., Ptito A. (2011). Electrophysiology and functional MRI in post-acute mild traumatic brain injury. J. Neurotrauma.

[195] Gosselin N., Bottari C., Chen J.-K., Huntgeburth S.C., de Beaumont L., Petrides M., Cheung B., Ptito A. (2012). Evaluating the cognitive consequences of mild traumatic brain injury and concussion by using electrophysiology. Neurosurg. Focus.

[196] Ozen L.J., Itier R.J., Preston F.F., Fernandes M.A. (2013). Long-term working memory deficits after concussion: Electrophysiological evidence. Brain Inj..

[197] Connolly M., Vespa P., Pouratian N., Gonzalez N.R., Hu X. (2015). Characterization of the relationship between intracranial pressure and electroencephalographic monitoring in burst-suppressed patients. Neurocrit. Care.

[198] Hartings J.A., Wilson J.A., Hinzman J.M., Pollandt S., Dreier J.P., DiNapoli V., Ficker D.M., Shutter L.A., Andaluz N. (2014). Spreading depression in continuous electroencephalography of brain trauma. Ann. Neurol..

[199] Munivenkatappa A., Rajeswaran J., Indira Devi B., Bennet N., Upadhyay N. (2014). EEG Neurofeedback therapy: Can it attenuate brain changes in TBI?. NeuroRehabilitation.

[200] Anderzhanova E., Wotjak C.T. (2013). Brain microdialysis and its applications in experimental neurochemistry. Cell Tissue Res..

[201] Hillered L., Dahlin A.P., Clausen F., Chu J., Bergquist J., Hjort K., Enblad P., Lewén A. (2014). Cerebral microdialysis for protein biomarker monitoring in the neurointensive care setting a technical approach. Front. Neurol..

[202] Hertle D.N., Santos E., Hagenston A.M., Jungk C., Haux D., Unterberg A.W., Sakowitz O.W. (2014). Cerebral glucose metabolism and sedation in brain-injured patients: A microdialysis study. J. Neurosurg. Anesthesiol..

[203] Kitagawa R., Yokobori S., Mazzeo A.T., Bullock R. (2013). Microdialysis in the neurocritical care unit. Neurosurg. Clin. N. Am..

[204] Rostami E. (2014). Glucose and the injured brain-monitored in the neurointensive care unit. Front. Neurol..

[205] Thelin E.P., Nelson D.W., Ghatan P.H., Bellander B.-M. (2014). Microdialysis monitoring of CSF parameters in severe traumatic brain injury patients: A novel approach. Front. Neurol..

[206] Dienel G.A. (2014). Lactate shuttling and lactate use as fuel after traumatic brain injury: Metabolic considerations. J. Cereb. Blood Flow Metab..

[207] Jalloh I., Helmy A., Shannon R.J., Gallagher C.N., Menon D.K., Carpenter K.L.H., Hutchinson P.J. (2013). Lactate uptake by the injured human brain: Evidence from an arteriovenous gradient and cerebral microdialysis study. J. Neurotrauma.

[208] Bouzat P., Oddo M., Payen J.-F. (2014). Transcranial Doppler after traumatic brain injury: Is there a role?. Curr. Opin. Crit. Care.

[209] Sahuquillo J., Merino M.-A., Sánchez-Guerrero A., Arikan F., Vidal-Jorge M., Martínez-Valverde T., Rey A., Riveiro M., Poca M.-A. (2014). Lactate and the lactate-to-pyruvate molar ratio cannot be used as independent biomarkers for monitoring brain energetic metabolism: A microdialysis study in patients with traumatic brain injuries. PLoS ONE.

[210] Bouzat P., Oddo M. (2014). Lactate and the injured brain: Friend or foe?. Curr. Opin. Crit. Care.

[211] Carre E., Ogier M., Boret H., Montcriol A., Bourdon L., Jean-Jacques R. (2013). Metabolic crisis in severely head-injured patients: Is ischemia just the tip of the iceberg?. Front. Neurol..

[212] Blyth B.J., Farhavar A., Gee C., Hawthorn B., He H., Nayak A., Stöcklein V., Bazarian J.J. (2009). Validation of serum markers for blood-brain barrier disruption in traumatic brain injury. J. Neurotrauma.

[213] Babu R., Bagley J.H., Di C., Friedman A.H., Adamson C. (2012). Thrombin and hemin as central factors in the mechanisms of intracerebral hemorrhage-induced secondary brain injury and as potential targets for intervention. Neurosurg. Focus.

[214] Park U.J., Lee Y.A., Won S.M., Lee J.H., Kang S.-H., Springer J.E., Lee Y.B., Gwag B.J. (2011). Blood-derived iron mediates free radical production and neuronal death in the hippocampal CA1 area following transient forebrain ischemia in rat. Acta Neuropathol. (Berl.).

[215] Bazarian J.J., Beck C., Blyth B., von Ahsen N., Hasselblatt M. (2006). Impact of creatine kinase correction on the predictive value of S-100B after mild traumatic brain injury. Restor. Neurol. Neurosci..

[216] Liu Y., Cai H., Wang Z., Li J., Wang K., Yu Z., Chen G. (2013). Induction of autophagy by cystatin C: A potential mechanism for prevention of cerebral vasospasm after experimental subarachnoid hemorrhage. Eur. J. Med. Res..

[217] Mahley R.W., Huang Y. (2012). Apolipoprotein e sets the stage: Response to injury triggers neuropathology. Neuron.

[218] Wang H., Sama A.E. (2012). Anti-inflammatory role of Fetuin-A in injury and infection. Curr. Mol. Med..

[219] Ducruet A.F., Zacharia B.E., Hickman Z.L., Grobelny B.T., Yeh M.L., Sosunov S.A., Connolly E.S. (2009). The complement cascade as a therapeutic target in intracerebral hemorrhage. Exp. Neurol..

[220] Khanna A., Kahle K.T., Walcott B.P., Gerzanich V., Simard J.M. (2014). Disruption of ion homeostasis in the neurogliovascular unit underlies the pathogenesis of ischemic cerebral edema. Transl. Stroke Res..

[221] Song M., Yu S.P. (2014). Ionic regulation of cell volume changes and cell death after ischemic stroke. Transl. Stroke Res..

[222] Sun D., Kahle K.T. (2014). Dysregulation of diverse Ion transport pathways controlling cell volume homoestasis contribute to neuroglial cell injury following ischemic stroke. Transl. Stroke Res..

[223] Martínez-Valverde T., Vidal-Jorge M., Montoya N., Sánchez-Guerrero A., Manrique S., Munar F., Pellegri M.-D., Poca M.-A., Sahuquillo J. (2015). Brain microdialysis as a tool to explore the ionic profile of the brain extracellular space in neurocritical patients: A methodological approach and feasibility study. J. Neurotrauma.

[224] Shah N.H., Aizenman E. (2014). Voltage-gated potassium channels at the crossroads of neuronal function, ischemic tolerance, and neurodegeneration. Transl. Stroke Res..

[225] Antunes A.P., Schiefecker A.J., Beer R., Pfausler B., Sohm F., Fischer M., Dietmann A., Lackner P., Hackl W.O., Ndayisaba J.-P. (2014). Higher brain extracellular potassium is associated with brain metabolic distress and poor outcome after aneurysmal subarachnoid hemorrhage. Crit. Care Lond. Engl..

[226] Dahlin A.P., Purins K., Clausen F., Chu J., Sedigh A., Lorant T., Enblad P., Lewén A., Hillered L. (2014). Refined microdialysis method for protein biomarker sampling in acute brain injury in the neurointensive care setting. Anal. Chem..

[227] Azeredo F.J., Dalla Costa T., Derendorf H. (2014). Role of microdialysis in pharmacokinetics and pharmacodynamics: Current status and future directions. Clin. Pharmacokinet..

[228] Bilgin-Freiert A., Dusick J.R., Stein N.R., Etchepare M., Vespa P., Gonzalez N.R. (2012). Muscle microdialysis to confirm sublethal ischemia in the induction of remote ischemic preconditioning. Transl. Stroke Res..

[229] Dollé J.-P., Morrison B., Schloss R.R., Yarmush M.L. (2013). An organotypic uniaxial strain model using microfluidics. Lab. Chip.

[230] Dollé J.-P., Morrison B., Schloss R.S., Yarmush M.L. (2014). Brain-on-a-chip microsystem for investigating traumatic brain injury: Axon diameter and mitochondrial membrane changes play a significant role in axonal response to strain injuries. Technology.

[231] Yu S.P., Wei Z., Wei L. (2013). Preconditioning strategy in stem cell transplantation therapy. Transl. Stroke Res..

[232] Yamauchi T., Saito H., Ito M., Shichinohe H., Houkin K., Kuroda S. (2014). Platelet lysate and granulocyte-colony stimulating factor serve safe and accelerated expansion of human bone marrow stromal cells for stroke therapy. Transl. Stroke Res..

[233] Chen D., Yu S.P., Wei L. (2014). Ion channels in regulation of neuronal regenerative activities. Transl. Stroke Res..

[234] Tajiri N., Kaneko Y., Shinozuka K., Ishikawa H., Yankee E., McGrogan M., Case C., Borlongan C.V. (2013). Stem cell recruitment of newly formed host cells via a successful seduction? filling the gap between neurogenic niche and injured brain site. PLoS ONE.

[235] Badaut J., Bix G.J. (2014). Vascular neural network phenotypic transformation after traumatic injury: Potential role in long-term sequelae. Transl. Stroke Res..

[236] Pati S., Khakoo A.Y., Zhao J., Jimenez F., Gerber M.H., Harting M., Redell J.B., Grill R., Matsuo Y., Guha S. (2011). Human mesenchymal stem cells inhibit vascular permeability by modulating vascular endothelial cadherin/β-catenin signaling. Stem Cells Dev..

[237] Springer J.E. (2002). Apoptotic cell death following traumatic injury to the central nervous system. J. Biochem. Mol. Biol..

[238] Li H., Gao A., Feng D., Wang Y., Zhang L., Cui Y., Li B., Wang Z., Chen G. (2014). Evaluation of the protective potential of brain microvascular endothelial cell autophagy on blood–brain barrier Integrity during experimental cerebral ischemia–reperfusion injury. Transl. Stroke Res..

[239] Torrente D., Avila M., Cabezas R., Morales L., Gonzalez J., Samudio I., Barreto G. (2014). Paracrine factors of human mesenchymal stem cells increase wound closure and reduce reactive oxygen species production in a traumatic brain injury *in vitro* model. Hum. Exp. Toxicol..

[240] Hughes R.H., Silva V.A., Ahmed I., Shreiber D.I., Morrison B. (2014). Neuroprotection by genipin against reactive oxygen and reactive nitrogen species-mediated injury in organotypic hippocampal slice cultures. Brain Res..

[241] Hetz R.A., Bedi S.S., Olson S., Olsen A., Cox C.S. (2012). Progenitor cells: Therapeutic targets after traumatic brain injury. Transl. Stroke Res..

[242] Galindo L.T., Filippo T.R.M., Semedo P., Ariza C.B., Moreira C.M., Camara N.O.S., Porcionatto M.A. (2011). Mesenchymal stem cell therapy modulates the inflammatory response in experimental traumatic brain injury. Neurol. Res. Int..

[243] Iser I.C., Bracco P.A., Gonçalves C.E.I., Zanin R.F., Nardi N.B., Lenz G., Battastini A.M.O., Wink M.R. (2014). Mesenchymal stem cells from different murine tissues have differential capacity to metabolize extracellular nucleotides. J. Cell. Biochem..

[244] Wang J., Liao L., Wang S., Tan J. (2013). Cell therapy with autologous mesenchymal stem cells-how the disease process impacts clinical considerations. Cytotherapy.

[245] Chen B.-Y., Wang X., Chen L.-W., Luo Z.-J. (2012). Molecular targeting regulation of proliferation and differentiation of the bone marrow-derived mesenchymal stem cells or mesenchymal stromal cells. Curr. Drug Targets.

[246] Zhang R., Liu Y., Yan K., Chen L., Chen X.-R., Li P., Chen F.-F., Jiang X.-D. (2013). Anti-inflammatory and immunomodulatory mechanisms of mesenchymal stem cell transplantation in experimental traumatic brain injury. J. Neuroinflamm..

[247] Bedi S.S., Hetz R., Thomas C., Smith P., Olsen A.B., Williams S., Xue H., Aroom K., Uray K., Hamilton J. (2013). Intravenous multipotent adult progenitor cell therapy attenuates activated microglial/macrophage response and improves spatial learning after traumatic brain injury. Stem Cells Transl. Med..

[248] Park K.J., Park E., Liu E., Baker A.J. (2014). Bone marrow-derived endothelial progenitor cells protect postischemic axons after traumatic brain injury. J. Cereb. Blood Flow Metab..

[249] Spahn D.R., Waschke K.F., Standl T., Motsch J., van Huynegem L., Welte M., Gombotz H., Coriat P., Verkh L., Faithfull S. (2002). Use of perflubron emulsion to decrease allogeneic blood transfusion in high-blood-loss non-cardiac surgery: Results of a European phase 3 study. Anesthesiology.

[250] Sakas D.E., Crowell R.M., Kim K., Korosue K., Zervas N.T. (1994). The perfluorocarbon fluoromethyloadamantane offers cerebral protection in a model of isovolemic hemodilution in rabbits. Stroke J. Cereb. Circ..

[251] Daugherty W.P., Levasseur J.E., Sun D., Spiess B.D., Bullock M.R. (2004). Perfluorocarbon emulsion improves cerebral oxygenation and mitochondrial function after fluid percussion brain injury in rats. Neurosurgery.

[252] Berlin J.M., Leonard A.D., Pham T.T., Sano D., Marcano D.C., Yan S., Fiorentino S., Milas Z.L., Kosynkin D.V., Price B.K. (2010). Effective drug delivery, *in vitro* and *in vivo*, by carbon-based nanovectors noncovalently loaded with unmodified Paclitaxel. ACS Nano.

[253] Marcano D.C., Bitner B.R., Berlin J.M., Jarjour J., Lee J.M., Jacob A., Fabian R.H., Kent T.A., Tour J.M. (2013). Design of poly(ethylene glycol)-functionalized hydrophilic carbon clusters for targeted therapy of cerebrovascular dysfunction in mild traumatic brain injury. J. Neurotrauma.

[254] Yokobori S., Mazzeo A.T., Hosein K., Gajavelli S., Dietrich W.D., Bullock M.R. (2013). Preconditioning for traumatic brain injury. Transl. Stroke Res..

[255] Chen Z., Ni P., Lin Y., Xiao H., Chen J., Qian G., Ye Y., Xu S., Wang J., Yang X. (2010). Visual pathway lesion and its development during hyperbaric oxygen treatment: A bold- fMRI and DTI study. J. Magn. Reson. Imaging.

[256] Huang L., Obenaus A. (2011). Hyperbaric oxygen therapy for traumatic brain injury. Med. Gas Res..

[257] Neubauer R.A., James P. (1998). Cerebral oxygenation and the recoverable brain. Neurol. Res..

[258] Vlodavsky E., Palzur E., Soustiel J.F. (2006). Hyperbaric oxygen therapy reduces neuroinflammation and expression of matrix metalloproteinase-9 in the rat model of traumatic brain injury. Neuropathol. Appl. Neurobiol..

[259] Lin K.-C., Niu K.-C., Tsai K.-J., Kuo J.-R., Wang L.-C., Chio C.-C., Chang C.-P. (2012). Attenuating inflammation but stimulating both angiogenesis and neurogenesis using hyperbaric oxygen in rats with traumatic brain injury. J. Trauma Acute Care Surg..

[260] Zhang J.H., Lo T., Mychaskiw G., Colohan A. (2005). Mechanisms of hyperbaric oxygen and neuroprotection in stroke. Pathophysiology.

[261] Xiong L., Zhu Z., Dong H., Hu W., Hou L., Chen S. (2000). Hyperbaric oxygen preconditioning induces neuroprotection against ischemia in transient not permanent middle cerebral artery occlusion rat model. Chin. Med. J. (Engl.).

[262] Qin Z., Song S., Xi G., Silbergleit R., Keep R.F., Hoff J.T., Hua Y. (2007). Preconditioning with hyperbaric oxygen attenuates brain edema after experimental intracerebral hemorrhage. Neurosurg. Focus.

[263] Wang G.-H., Zhang X.-G., Jiang Z.-L., Li X., Peng L.-L., Li Y.-C., Wang Y. (2010). Neuroprotective effects of hyperbaric oxygen treatment on traumatic brain injury in the rat. J. Neurotrauma.

[264] Harch P.G., Kriedt C., Van Meter K.W., Sutherland R.J. (2007). Hyperbaric oxygen therapy improves spatial learning and memory in a rat model of chronic traumatic brain injury. Brain Res..

[265] Harch P.G., Andrews S.R., Fogarty E.F., Amen D., Pezzullo J.C., Lucarini J., Aubrey C., Taylor D.V., Staab P.K., van Meter K.W. (2012). A phase I study of low-pressure hyperbaric oxygen therapy for blast-induced post-concussion syndrome and post-traumatic stress disorder. J. Neurotrauma.

[266] Gertsch J.H., Seto T.B., Mor J., Onopa J. (2002). Ginkgo biloba for the prevention of severe acute mountain sickness (AMS) starting one day before rapid ascent. High Alt. Med. Biol..

[267] Gallagher S.A., Hackett P.H. (2004). High-altitude illness. Emerg. Med. Clin. North Am..

[268] Stoller K.P. (2011). Hyperbaric oxygen therapy (1.5 ATA) in treating sports related TBI/CTE: Two case reports. Med. Gas Res..

[269] Raghupathi R. (2004). Cell death mechanisms following traumatic brain injury. Brain Pathol. Zurich Switz..

[270] Vandresen-Filho S., Hoeller A.A., Herculano B.A., Duzzioni M., Duarte F.S., Piermartiri T.C.B., Boeck C.C., de Lima T.C.M., Marino-Neto J., Tasca C.I. (2013). NMDA preconditioning attenuates cortical and hippocampal seizures induced by intracerebroventricular quinolinic acid infusion. Neurotox. Res..

[271] Constantino L.C. (2014). The role of NMDA receptors in the development of brain resistance through pre- and postconditioning. Aging Dis..

[272] Chuang D.M., Gao X.M., Paul S.M. (1992). *N*-methyl-D-aspartate exposure blocks glutamate toxicity in cultured cerebellar granule cells. Mol. Pharmacol..

[273] Soriano F.X., Papadia S., Hofmann F., Hardingham N.R., Bading H., Hardingham G.E. (2006). Preconditioning doses of NMDA promote neuroprotection by enhancing neuronal excitability. J. Neurosci..

[274] Muir K.W., Lees K.R. (1995). Clinical experience with excitatory amino acid antagonist drugs. Stroke J. Cereb. Circ..

[275] Choi D.W. (1988). Glutamate neurotoxicity and diseases of the nervous system. Neuron.

[276] Boeck C.R., Ganzella M., Lottermann A., Vendite D. (2004). NMDA preconditioning protects against seizures and hippocampal neurotoxicity induced by quinolinic acid in mice. Epilepsia.

[277] Boeck C.R., Carbonera L.S., Milioli M.E., Constantino L.C., Garcez M.L., Rezin G.T., Scaini G., Streck E.L. (2013). Mitochondrial respiratory chain and creatine kinase activities following trauma brain injury in brain of mice preconditioned with N-methyl-d-aspartate. Mol. Cell. Biochem..

[278] Lin F.K., Suggs S., Lin C.H., Browne J.K., Smalling R., Egrie J.C., Chen K.K., Fox G.M., Martin F., Stabinsky Z. (1985). Cloning and expression of the human erythropoietin gene. Proc. Natl. Acad. Sci. USA.

[279] Anagnostou A., Liu Z., Steiner M., Chin K., Lee E.S., Kessimian N., Noguchi C.T. (1994). Erythropoietin receptor mRNA expression in human endothelial cells. Proc. Natl. Acad. Sci. USA.

[280] Genc S., Koroglu T.F., Genc K. (2004). Erythropoietin as a novel neuroprotectant. Restor. Neurol. Neurosci..

[281] Maiese K., Li F., Chong Z.Z. (2005). New avenues of exploration for erythropoietin. JAMA.

[282] Souvenir R., Flores J.J., Ostrowski R.P., Manaenko A., Duris K., Tang J. (2014). Erythropoietin inhibits HIF-1α expression via upregulation of PHD-2 transcription and translation in an *in vitro* model of hypoxia–ischemia. Transl. Stroke Res..

[283] Wang L., Zhang Z., Wang Y., Zhang R., Chopp M. (2004). Treatment of stroke with erythropoietin enhances neurogenesis and angiogenesis and improves neurological function in rats. Stroke J. Cereb. Circ..

[284] Ribatti D., Vacca A., Roccaro A.M., Crivellato E., Presta M. (2003). Erythropoietin as an angiogenic factor. Eur. J. Clin. Investig..

[285] Marti H.H., Bernaudin M., Petit E., Bauer C. (2000). Neuroprotection and angiogenesis: Dual role of erythropoietin in brain ischemia. Physiology.

[286] Jaquet K., Krause K., Tawakol-Khodai M., Geidel S., Kuck K.-H. (2002). Erythropoietin and VEGF exhibit equal angiogenic potential. Microvasc. Res..

[287] Chong Z.Z., Kang J.-Q., Maiese K. (2002). Hematopoietic factor erythropoietin fosters neuroprotection through novel signal transduction cascades. J. Cereb. Blood Flow Metab..

[288] Maiese K., Chong Z.Z. (2004). Insights into oxidative stress and potential novel therapeutic targets for Alzheimer disease. Restor. Neurol. Neurosci..

[289] Sakamaki K. (2004). Regulation of endothelial cell death and its role in angiogenesis and vascular regression. Curr. Neurovasc. Res..

[290] Cotena S., Piazza O., Tufano R. (2008). The use of erythtropoietin in cerebral diseases. Panminerva Med..

[291] Velly L., Pellegrini L., Guillet B., Bruder N., Pisano P. (2010). Erythropoietin 2nd cerebral protection after acute injuries: A double-edged sword?. Pharmacol. Ther..

[292] Yatsiv I., Grigoriadis N., Simeonidou C., Stahel P.F., Schmidt O.I., Alexandrovitch A.G., Tsenter J., Shohami E. (2005). Erythropoietin is neuroprotective, improves functional recovery, and reduces neuronal apoptosis and inflammation in a rodent model of experimental closed head injury. FASEB J..

[293] Brines M.L., Ghezzi P., Keenan S., Agnello D., de Lanerolle N.C., Cerami C., Itri L.M., Cerami A. (2000). Erythropoietin crosses the blood-brain barrier to protect against experimental brain injury. Proc. Natl. Acad. Sci. USA.

[294] Ozturk E., Demirbilek S., Kadir But A., Saricicek V., Gulec M., Akyol O., Ozcan Ersoy M. (2005). Antioxidant properties of propofol and erythropoietin after closed head injury in rats. Prog. Neuropsychopharmacol. Biol. Psychiatry.

[295] Yildirim E., Ozisik K., Solaroglu I., Kaptanoglu E., Beskonakli E., Sargon M.F., Kilinc K., Sakinci U. (2005). Protective effect of erythropoietin on type II pneumocyte cells after traumatic brain injury in rats. J. Trauma.

[296] Wang L., Wang X., Su H., Han Z., Yu H., Wang D., Jiang R., Liu Z., Zhang J. (2015). Recombinant human erythropoietin improves the neurofunctional recovery of rats following traumatic brain injury via an increase in circulating endothelial progenitor cells. Transl. Stroke Res..

[297] Corwin H.L., Gettinger A., Fabian T.C., May A., Pearl R.G., Heard S., An R., Bowers P.J., Burton P., Klausner M.A. (2007). Efficacy and safety of epoetin alfa in critically ill patients. N. Engl. J. Med..

[298] Guennoun R., Labombarda F., Gonzalez Deniselle M.C., Liere P., de Nicola A.F., Schumacher M. (2015). Progesterone and allopregnanolone in the central nervous system: Response to injury and implication for neuroprotection. J. Steroid Biochem. Mol. Biol..

[299] Baulieu E.E., Schumacher M., Koenig H., Jung-Testas I., Akwa Y. (1996). Progesterone as a neurosteroid: Actions within the nervous system. Cell. Mol. Neurobiol..

[300] Wright D.W., Kellermann A.L., Hertzberg V.S., Clark P.L., Frankel M., Goldstein F.C., Salomone J.P., Dent L.L., Harris O.A., Ander D.S. (2007). ProTECT: A Randomized Clinical Trial of Progesterone for Acute Traumatic Brain Injury. Ann. Emerg. Med..

[301] Xiao G., Wei J., Yan W., Wang W., Lu Z. (2008). Improved outcomes from the administration of progesterone for patients with acute severe traumatic brain injury: A randomized controlled trial. Crit. Care Lond. Engl..

[302] Chase A. (2015). Traumatic brain injury: No benefit of progesterone therapy in patients with TBI. Nat. Rev. Neurol..

[303] Skolnick B.E., Maas A.I., Narayan R.K., van der Hoop R.G., MacAllister T., Ward J.D., Nelson N.R., Stocchetti N. (2014). A clinical trial of progesterone for severe traumatic brain injury. N. Engl. J. Med..

[304] Margulies S., Hicks R. (2009). Combination Therapies for Traumatic Brain Injury Workshop Leaders Combination therapies for traumatic brain injury: Prospective considerations. J. Neurotrauma.

[305] Narayan R.K., Michel M.E., Ansell B., Baethmann A., Biegon A., Bracken M.B., Bullock M.R., Choi S.C., Clifton G.L., Contant C.F. (2002). Clinical trials in head injury. J. Neurotrauma.

[306] Mustafa A.G., Singh I.N., Wang J., Carrico K.M., Hall E.D. (2010). Mitochondrial protection after traumatic brain injury by scavenging lipid peroxyl radicals. J. Neurochem..

[307] Mustafa A.G., Wang J.A., Carrico K.M., Hall E.D. (2011). Pharmacological inhibition of lipid peroxidation attenuates calpain-mediated cytoskeletal degradation after traumatic brain injury. J. Neurochem..

[308] Reiter R.J., Carneiro R.C., Oh C.S. (1997). Melatonin in relation to cellular antioxidative defense mechanisms. Horm. Metab. Res..

[309] Samantaray S., Das A., Thakore N.P., Matzelle D.D., Reiter R.J., Ray S.K., Banik N.L. (2009). Therapeutic potential of melatonin in traumatic central nervous system injury. J. Pineal Res..

[310] Ozdemir D., Tugyan K., Uysal N., Sonmez U., Sonmez A., Acikgoz O., Ozdemir N., Duman M., Ozkan H. (2005). Protective effect of melatonin against head trauma-induced hippocampal damage and spatial memory deficits in immature rats. Neurosci. Lett..

[311] Ozdemir D., Uysal N., Gonenc S., Acikgoz O., Sonmez A., Topcu A., Ozdemir N., Duman M., Semin I., Ozkan H. (2005). Effect of melatonin on brain oxidative damage induced by traumatic brain injury in immature rats. Physiol. Res. Acad. Sci. Bohemoslov..

[312] Beni S.M., Kohen R., Reiter R.J., Tan D.-X., Shohami E. (2004). Melatonin-induced neuroprotection after closed head injury is associated with increased brain antioxidants and attenuated late-phase activation of NF-κB and AP-1. FASEB J..

[313] Xiong Y., Singh I.N., Hall E.D. (2009). Tempol protection of spinal cord mitochondria from peroxynitrite-induced oxidative damage. Free Radic. Res..

[314] Hillard V.H., Peng H., Zhang Y., Das K., Murali R., Etlinger J.D., Zeman R.J. (2004). Tempol, a nitroxide antioxidant, improves locomotor and histological outcomes after spinal cord contusion in rats. J. Neurotrauma.

[315] Wilcox C.S. (2010). Effects of tempol and redox-cycling nitroxides in models of oxidative stress. Pharmacol. Ther..

[316] Carroll R.T., Galatsis P., Borosky S., Kopec K.K., Kumar V., Althaus J.S., Hall E.D. (2000). 4-Hydroxy-2,2,6,6-tetramethylpiperidine-1-oxyl (Tempol) inhibits peroxynitrite-mediated phenol nitration. Chem. Res. Toxicol..

[317] Deng-Bryant Y., Singh I.N., Carrico K.M., Hall E.D. (2008). Neuroprotective effects of tempol, a catalytic scavenger of peroxynitrite-derived free radicals, in a mouse traumatic brain injury model. J. Cereb. Blood Flow Metab..

[318] Hall E.D., Vaishnav R.A., Mustafa A.G. (2010). Antioxidant therapies for traumatic brain injury. Neurother. J. Am. Soc. Exp. Neurother..

[319] Singleton R.H., Yan H.Q., Fellows-Mayle W., Dixon C.E. (2010). Resveratrol attenuates behavioral impairments and reduces cortical and hippocampal loss in a rat controlled cortical impact model of traumatic brain injury. J. Neurotrauma.

[320] Sönmez U., Sönmez A., Erbil G., Tekmen I., Baykara B. (2007). Neuroprotective effects of resveratrol against traumatic brain injury in immature rats. Neurosci. Lett..

[321] Ates O., Cayli S., Altinoz E., Gurses I., Yucel N., Sener M., Kocak A., Yologlu S. (2007). Neuroprotection by resveratrol against traumatic brain injury in rats. Mol. Cell. Biochem..

[322] Mendes Arent A., de Souza L.F., Walz R., Dafre A.L. (2014). Perspectives on molecular biomarkers of oxidative stress and antioxidant strategies in traumatic brain injury. BioMed Res. Int..

[323] Andrews P.J.D., Sinclair H.L., Battison C.G., Polderman K.H., Citerio G., Mascia L., Harris B.A., Murray G.D., Stocchetti N., Menon D.K. (2011). European society of intensive care medicine study of therapeutic hypothermia (32–35 °C) for intracranial pressure reduction after traumatic brain injury (the Eurotherm3235Trial). Trials.

[324] Price T., McGloin S. (2003). A review of cooling patients with severe cerebral insult in ICU (Part 1). Nurs. Crit. Care.

[325] Harris B., Andrews P., Murray G., Forbes J., Moseley O. (2012). Systematic review of head cooling in adults after traumatic brain injury and stroke. Health Technol. Assess..

[326] Chesnut R.M., Marshall L.F., Klauber M.R., Blunt B.A., Baldwin N., Eisenberg H.M., Jane J.A., Marmarou A., Foulkes M.A. (1993). The role of secondary brain injury in determining outcome from severe head injury. J. Trauma.

[327] Liu W.G., Qiu W.S., Zhang Y., Wang W.M., Lu F., Yang X.F. (2006). Effects of selective brain cooling in patients with severe traumatic brain injury: A preliminary study. J. Int. Med. Res..

[328] Patterson J., Bloom S.A., Coyle B., Mouradjian D., Wilensky E.M. (2005). Successful outcome in severe traumatic brain injury: A case study. J. Neurosci. Nurs..

[329] Dings J., Meixensberger J., Jäger A., Roosen K. (1998). Clinical experience with 118 brain tissue oxygen partial pressure catheter probes. Neurosurgery.

[330] Kiening K.L., Unterberg A.W., Bardt T.F., Schneider G.H., Lanksch W.R. (1996). Monitoring of cerebral oxygenation in patients with severe head injuries: Brain tissue PO2 *vs.* jugular vein oxygen saturation. J. Neurosurg..

[331] Meixensberger J., Jaeger M., Väth A., Dings J., Kunze E., Roosen K. (2003). Brain tissue oxygen guided treatment supplementing ICP/CPP therapy after traumatic brain injury. J. Neurol. Neurosurg. Psychiatry.

[332] Sakurai Y., Shima M., Matsumoto T., Takatsuka H., Nishiya K., Kasuda S., Fujimura Y., Yoshioka A. (2003). Anticoagulant activity of M-LAO, L-amino acid oxidase purified from Agkistrodon halys blomhoffii, through selective inhibition of factor IX. Biochim. Biophys. Acta.

[333] Polderman K.H. (2004). Application of therapeutic hypothermia in the ICU: Opportunities and pitfalls of a promising treatment modality. Part 1: Indications and evidence. Intensive Care Med..

[334] Polderman K.H. (2004). Application of therapeutic hypothermia in the intensive care unit. Opportunities and pitfalls of a promising treatment modality—Part 2: Practical aspects and side effects. Intensive Care Med..

[335] Tokutomi T., Morimoto K., Miyagi T., Yamaguchi S., Ishikawa K., Shigemori M. (2007). Optimal temperature for the management of severe traumatic brain injury: Effect of hypothermia on intracranial pressure, systemic and intracranial hemodynamics, and metabolism. Neurosurgery.

[336] Lee H.-C., Chuang H.-C., Cho D.-Y., Cheng K.-F., Lin P.-H., Chen C.-C. (2010). Applying cerebral hypothermia and brain oxygen monitoring in treating severe traumatic brain injury. World Neurosurg..

[337] Li Y., Zhang C., Zhang X., Zhou H., Meng L. (2015). Effects of mild induced hypothermia on hippocampal connexin 43 and glutamate transporter 1 expression following traumatic brain injury in rats. Mol. Med. Rep..

[338] Maier C.M., Ahern K.V., Cheng M.L., Lee J.E., Yenari M.A., Steinberg G.K. (1998). Optimal depth and duration of mild hypothermia in a focal model of transient cerebral ischemia: Effects on neurologic outcome, infarct size, apoptosis, and inflammation. Stroke J. Cereb. Circ..

[339] Sydenham E., Roberts I., Alderson P. (2009). Hypothermia for traumatic head injury. Cochrane Database Syst. Rev..

[340] Saxena M., Andrews P.J., Cheng A., Deol K., Hammond N. (2014). Modest cooling therapies (35 °C to 37.5 °C) for traumatic brain injury. Cochrane Database of Systematic Reviews.

[341] Guidelines for the management of severe traumatic brain injury, 3rd Edition. https://www.braintrauma.org/pdf/protected/Guidelines_Management_2007w_bookmarks.pdf.

[342] Moore E.M., Nichol A.D., Bernard S.A., Bellomo R. (2011). Therapeutic hypothermia: Benefits, mechanisms and potential clinical applications in neurological, cardiac and kidney injury. Injury.

[343] Bettayeb K., Oumata N., Echalier A., Ferandin Y., Endicott J.A., Galons H., Meijer L. (2008). CR8, a potent and selective, roscovitine-derived inhibitor of cyclin-dependent kinases. Oncogene.

[344] Taupin P. (2007). BrdU immunohistochemistry for studying adult neurogenesis: Paradigms, pitfalls, limitations, and validation. Brain Res. Rev..

[345] Cernak I., Stoica B., Byrnes K.R., di Giovanni S., Faden A.I. (2005). Role of the cell cycle in the pathobiology of central nervous system trauma. Cell Cycle Georget. Tex.

[346] Di Giovanni S., Movsesyan V., Ahmed F., Cernak I., Schinelli S., Stoica B., Faden A.I. (2005). Cell cycle inhibition provides neuroprotection and reduces glial proliferation and scar formation after traumatic brain injury. Proc. Natl. Acad. Sci. USA.

[347] Hilton G.D., Stoica B.A., Byrnes K.R., Faden A.I. (2008). Roscovitine reduces neuronal loss, glial activation, and neurologic deficits after brain trauma. J. Cereb. Blood Flow Metab..

[348] Kabadi S.V., Stoica B.A., Byrnes K.R., Hanscom M., Loane D.J., Faden A.I. (2012). Selective CDK inhibitor limits neuroinflammation and progressive neurodegeneration after brain trauma. J. Cereb. Blood Flow Metab..

[349] Kabadi S.V., Stoica B.A., Loane D.J., Byrnes K.R., Hanscom M., Cabatbat R.M., Tan M.T., Faden A.I. (2012). Cyclin D1 gene ablation confers neuroprotection in traumatic brain injury. J. Neurotrauma.

[350] Kabadi S.V., Stoica B.A., Hanscom M., Loane D.J., Kharebava G., Murray Ii M.G., Cabatbat R.M., Faden A.I. (2012). CR8, a selective and potent CDK inhibitor, provides neuroprotection in experimental traumatic brain injury. Neurotherapeutics.

[351] Wu J., Kharebava G., Piao C., Stoica B.A., Dinizo M., Sabirzhanov B., Hanscom M., Guanciale K., Faden A.I. (2012). Inhibition of E2F1/CDK1 pathway attenuates neuronal apoptosis *in vitro* and confers neuroprotection after spinal cord injury *in vivo*. PLoS ONE.

[352] Wu J., Pajoohesh-Ganji A., Stoica B.A., Dinizo M., Guanciale K., Faden A.I. (2012). Delayed expression of cell cycle proteins contributes to astroglial scar formation and chronic inflammation after rat spinal cord contusion. J. Neuroinflammation.

[353] Kabadi S.V., Stoica B.A., Loane D.J., Luo T., Faden A.I. (2014). CR8, a novel inhibitor of CDK, limits microglial activation, astrocytosis, neuronal loss, and neurologic dysfunction after experimental traumatic brain injury. J. Cereb. Blood Flow Metab..

[354] Chauhan N.B., Gatto R. (2010). Synergistic benefits of erythropoietin and simvastatin after traumatic brain injury. Brain Res..

[355] Abrahamson E.E., Ikonomovic M.D., Dixon C.E., DeKosky S.T. (2009). Simvastatin therapy prevents brain trauma-induced increases in beta-amyloid peptide levels. Ann. Neurol..

[356] Béziaud T., Ru Chen X., el Shafey N., Fréchou M., Teng F., Palmier B., Beray-Berthat V., Soustrat M., Margaill I., Plotkine M. (2011). Simvastatin in traumatic brain injury: Effect on brain edema mechanisms. Crit. Care Med..

[357] Chen G., Zhang S., Shi J., Ai J., Qi M., Hang C. (2009). Simvastatin reduces secondary brain injury caused by cortical contusion in rats: Possible involvement of TLR4/NF-κB pathway. Exp. Neurol..

[358] Chen X.R., Besson V.C., Beziaud T., Plotkine M., Marchand-Leroux C. (2008). Combination therapy with fenofibrate, a peroxisome proliferator-activated receptor alpha agonist, and simvastatin, a 3-hydroxy-3-methylglutaryl-coenzyme A reductase inhibitor, on experimental traumatic brain injury. J. Pharmacol. Exp. Ther..

[359] Indraswari F., Wang H., Lei B., James M.L., Kernagis D., Warner D.S., Dawson H.N., Laskowitz D.T. (2012). Statins improve outcome in murine models of intracranial hemorrhage and traumatic brain injury: A translational approach. J. Neurotrauma.

[360] Li B., Mahmood A., Lu D., Wu H., Xiong Y., Qu C., Chopp M. (2009). Simvastatin attenuates microglial cells and astrocyte activation and decreases interleukin-1beta level after traumatic brain injury. Neurosurgery.

[361] Reuter B., Rodemer C., Grudzenski S., Meairs S., Bugert P., Hennerici M.G., Fatar M. (2015). Effect of simvastatin on MMPs and TIMPs in human brain endothelial cells and experimental stroke. Transl. Stroke Res..

[362] Wang H., Lynch J.R., Song P., Yang H.-J., Yates R.B., Mace B., Warner D.S., Guyton J.R., Laskowitz D.T. (2007). Simvastatin and atorvastatin improve behavioral outcome, reduce hippocampal degeneration, and improve cerebral blood flow after experimental traumatic brain injury. Exp. Neurol..

[363] Lu D., Qu C., Goussev A., Jiang H., Lu C., Schallert T., Mahmood A., Chen J., Li Y., Chopp M. (2007). Statins increase neurogenesis in the dentate gyrus, reduce delayed neuronal death in the hippocampal CA3 region, and improve spatial learning in rat after traumatic brain injury. J. Neurotrauma.

[364] Devaraj S., Chan E., Jialal I. (2006). Direct demonstration of an antiinflammatory effect of simvastatin in subjects with the metabolic syndrome. J. Clin. Endocrinol. Metab..

[365] Wu H., Jiang H., Lu D., Qu C., Xiong Y., Zhou D., Chopp M., Mahmood A. (2011). Induction of angiogenesis and modulation of vascular endothelial growth factor receptor-2 by simvastatin after traumatic brain injury. Neurosurgery.

[366] Diaz-Arrastia R., Kochanek P.M., Bergold P., Kenney K., Marx C.E., Grimes C.J.B., Loh L.Y., Adam L.G.E., Oskvig D., Curley K.C. (2014). Pharmacotherapy of traumatic brain injury: State of the science and the road forward: Report of the department of defense neurotrauma pharmacology workgroup. J. Neurotrauma.

[367] Cameron H.A., Woolley C.S., McEwen B.S., Gould E. (1993). Differentiation of newly born neurons and glia in the dentate gyrus of the adult rat. Neuroscience.

[368] Cernak I. (2010). The importance of systemic response in the pathobiology of blast-induced neurotrauma. Front. Neurol..

[369] Dalle Lucca J.J., Chavko M., Dubick M.A., Adeeb S., Falabella M.J., Slack J.L., McCarron R., Li Y. (2012). Blast-induced moderate neurotrauma (BINT) elicits early complement activation and tumor necrosis factor α (TNFα) release in a rat brain. J. Neurol. Sci..

[370] Gorbunov N.V., McFaul S.J., Januszkiewicz A., Atkins J.L. (2005). Pro-inflammatory alterations and status of blood plasma iron in a model of blast-induced lung trauma. Int. J. Immunopathol. Pharmacol..

[371] Koliatsos V.E., Cernak I., Xu L., Song Y., Savonenko A., Crain B.J., Eberhart C.G., Frangakis C.E., Melnikova T., Kim H. (2011). A mouse model of blast injury to brain: Initial pathological, neuropathological, and behavioral characterization. J. Neuropathol. Exp. Neurol..

[372] Svetlov S.I., Prima V., Kirk D.R., Gutierrez H., Curley K.C., Hayes R.L., Wang K.K.W. (2010). Morphologic and biochemical characterization of brain injury in a model of controlled blast overpressure exposure. J. Trauma.

[373] Readnower R.D., Chavko M., Adeeb S., Conroy M.D., Pauly J.R., McCarron R.M., Sullivan P.G. (2010). Increase in blood-brain barrier permeability, oxidative stress, and activated microglia in a rat model of blast-induced traumatic brain injury. J. Neurosci. Res..

[374] Valiyaveettil M., Alamneh Y., Oguntayo S., Wei Y., Wang Y., Arun P., Nambiar M.P. (2012). Regional specific alterations in brain acetylcholinesterase activity after repeated blast exposures in mice. Neurosci. Lett..

[375] Pavlov V.A., Tracey K.J. (2006). Controlling inflammation: The cholinergic anti-inflammatory pathway. Biochem. Soc. Trans..

[376] Pavlov V.A., Wang H., Czura C.J., Friedman S.G., Tracey K.J. (2003). The cholinergic anti-inflammatory pathway: A missing link in neuroimmunomodulation. Mol. Med. Camb. Mass.

[377] Pavlov V.A., Tracey K.J. (2005). The cholinergic anti-inflammatory pathway. Brain. Behav. Immun..

[378] Gorbunov N.V., Asher L.V., Ayyagari V., Atkins J.L. (2006). Inflammatory leukocytes and iron turnover in experimental hemorrhagic lung trauma. Exp. Mol. Pathol..

[379] Leeds P.R., Yu F., Wang Z., Chiu C.-T., Zhang Y., Leng Y., Linares G.R., Chuang D.-M. (2014). A new avenue for lithium: Intervention in traumatic brain injury. ACS Chem. Neurosci..

[380] Hashimoto K., Shimizu E., Iyo M. (2004). Critical role of brain-derived neurotrophic factor in mood disorders. Brain Res. Rev..

[381] Post R.M. (2007). Role of BDNF in bipolar and unipolar disorder: Clinical and theoretical implications. J. Psychiatr. Res..

[382] Angelucci F., Aloe L., Jiménez-Vasquez P., Mathé A.A. (2003). Lithium treatment alters brain concentrations of nerve growth factor, brain-derived neurotrophic factor and glial cell line-derived neurotrophic factor in a rat model of depression. Int. J. Neuropsychopharmacol..

[383] Angelucci F., Mathé A.A., Aloe L. (2004). Neurotrophic factors and CNS disorders: Findings in rodent models of depression and schizophrenia. Prog. Brain Res..

[384] Gama C.S., Andreazza A.C., Kunz M., Berk M., Belmonte-de-Abreu P.S., Kapczinski F. (2007). Serum levels of brain-derived neurotrophic factor in patients with schizophrenia and bipolar disorder. Neurosci. Lett..

[385] Frey B.N., Andreazza A.C., Rosa A.R., Martins M.R., Valvassori S.S., Réus G.Z., Hatch J.P., Quevedo J., Kapczinski F. (2006). Lithium increases nerve growth factor levels in the rat hippocampus in an animal model of mania. Behav. Pharmacol..

[386] Hellweg R., Lang U.E., Nagel M., Baumgartner A. (2002). Subchronic treatment with lithium increases nerve growth factor content in distinct brain regions of adult rats. Mol. Psychiatry.

[387] Mudò G., Jiang X.H., Timmusk T., Bindoni M., Belluardo N. (1996). Change in neurotrophins and their receptor mRNAs in the rat forebrain after status epilepticus induced by pilocarpine. Epilepsia.

[388] Thau-Zuchman O., Shohami E., Alexandrovich A.G., Leker R.R. (2010). Vascular endothelial growth factor increases neurogenesis after traumatic brain injury. J. Cereb. Blood Flow Metab..

[389] Thau-Zuchman O., Shohami E., Alexandrovich A.G., Leker R.R. (2012). Combination of vascular endothelial and fibroblast growth factor 2 for induction of neurogenesis and angiogenesis after traumatic brain injury. J. Mol. Neurosci..

[390] Thau-Zuchman O., Shohami E., Alexandrovich A.G., Leker R.R. (2012). Subacute treatment with vascular endothelial growth factor after traumatic brain injury increases angiogenesis and gliogenesis. Neuroscience.

[391] Chen G., Rajkowska G., Du F., Seraji-Bozorgzad N., Manji H.K. (2000). Enhancement of hippocampal neurogenesis by lithium. J. Neurochem..

[392] Son H., Yu I.T., Hwang S.-J., Kim J.S., Lee S.-H., Lee Y.-S., Kaang B.-K., Lee S.-H. (2003). Lithium enhances long-term potentiation independently of hippocampal neurogenesis in the rat dentate gyrus. J. Neurochem..

[393] Senatorov V.V., Ren M., Kanai H., Wei H., Chuang D.-M. (2004). Short-term lithium treatment promotes neuronal survival and proliferation in rat striatum infused with quinolinic acid, an excitotoxic model of Huntington’s disease. Mol. Psychiatry.

[394] Hashimoto R., Senatorov V., Kanai H., Leeds P., Chuang D.-M. (2003). Lithium stimulates progenitor proliferation in cultured brain neurons. Neuroscience.

[395] Wexler E.M., Geschwind D.H., Palmer T.D. (2008). Lithium regulates adult hippocampal progenitor development through canonical Wnt pathway activation. Mol. Psychiatry.

[396] Ekici M.A., Uysal O., Cikriklar H.I., Özbek Z., Turgut Cosan D., Baydemir C., Kazanci B., Hafizoğlu D. (2014). Effect of etanercept and lithium chloride on preventing secondary tissue damage in rats with experimental diffuse severe brain injury. Eur. Rev. Med. Pharmacol. Sci..

[397] Yu F., Zhang Y., Chuang D.-M. (2012). Lithium reduces BACE1 overexpression, β amyloid accumulation, and spatial learning deficits in mice with traumatic brain injury. J. Neurotrauma.

[398] Cai H., Wang Y., McCarthy D., Wen H., Borchelt D.R., Price D.L., Wong P.C. (2001). BACE1 is the major beta-secretase for generation of Abeta peptides by neurons. Nat. Neurosci..

[399] Blasko I., Beer R., Bigl M., Apelt J., Franz G., Rudzki D., Ransmayr G., Kampfl A., Schliebs R. (2004). Experimental traumatic brain injury in rats stimulates the expression, production and activity of Alzheimer’s disease beta-secretase (BACE-1). J. Neural Transm..

[400] Loane D.J., Pocivavsek A., Moussa C.E.-H., Thompson R., Matsuoka Y., Faden A.I., Rebeck G.W., Burns M.P. (2009). Amyloid precursor protein secretases as therapeutic targets for traumatic brain injury. Nat. Med..

[401] Uryu K., Chen X.-H., Martinez D., Browne K.D., Johnson V.E., Graham D.I., Lee V.M.-Y., Trojanowski J.Q., Smith D.H. (2007). Multiple proteins implicated in neurodegenerative diseases accumulate in axons after brain trauma in humans. Exp. Neurol..

[402] Chuang D.-M., Leng Y., Marinova Z., Kim H.-J., Chiu C.-T. (2009). Multiple roles of HDAC inhibition in neurodegenerative conditions. Trends Neurosci..

[403] Dash P.K., Orsi S.A., Moore A.N. (2009). Histone deactylase inhibition combined with behavioral therapy enhances learning and memory following traumatic brain injury. Neuroscience.

[404] Shein N.A., Grigoriadis N., Alexandrovich A.G., Simeonidou C., Lourbopoulos A., Polyzoidou E., Trembovler V., Mascagni P., Dinarello C.A., Shohami E. (2009). Histone deacetylase inhibitor ITF2357 is neuroprotective, improves functional recovery, and induces glial apoptosis following experimental traumatic brain injury. FASEB J..

[405] Zhang B., West E.J., Van K.C., Gurkoff G.G., Zhou J., Zhang X.-M., Kozikowski A.P., Lyeth B.G. (2008). HDAC inhibitor increases histone H3 acetylation and reduces microglia inflammatory response following traumatic brain injury in rats. Brain Res..

[406] Yu F., Wang Z., Tanaka M., Chiu C.-T., Leeds P., Zhang Y., Chuang D.-M. (2013). Posttrauma cotreatment with lithium and valproate: Reduction of lesion volume, attenuation of blood-brain barrier disruption, and improvement in motor coordination in mice with traumatic brain injury. J. Neurosurg..

[407] Wang Z., Leng Y., Tsai L.-K., Leeds P., Chuang D.-M. (2011). Valproic acid attenuates blood-brain barrier disruption in a rat model of transient focal cerebral ischemia: The roles of HDAC and MMP-9 inhibition. J. Cereb. Blood Flow Metab..

[408] Yu F., Wang Z., Tchantchou F., Chiu C.-T., Zhang Y., Chuang D.-M. (2012). Lithium ameliorates neurodegeneration, suppresses neuroinflammation, and improves behavioral performance in a mouse model of traumatic brain injury. J. Neurotrauma.

[409] Chiu C.-T., Wang Z., Hunsberger J.G., Chuang D.-M. (2013). Therapeutic potential of mood stabilizers lithium and valproic acid: Beyond bipolar disorder. Pharmacol. Rev..

[410] Zhang Y.P., Cai J., Shields L.B.E., Liu N., Xu X.-M., Shields C.B. (2014). Traumatic brain injury using mouse models. Transl. Stroke Res..

[411] Yu G.-X., Mueller M., Hawkins B.E., Mathew B.P., Parsley M.A., Vergara L.A., Hellmich H.L., Prough D.S., DeWitt D.S. (2014). Traumatic Brain Injury *in Vivo* and *in Vitro* Contributes to Cerebral Vascular Dysfunction through Impaired Gap Junction Communication between Vascular Smooth Muscle Cells. J. Neurotrauma.

